# Ablation of the deubiquitinase USP15 ameliorates nonalcoholic fatty liver disease and nonalcoholic steatohepatitis

**DOI:** 10.1038/s12276-023-01036-7

**Published:** 2023-07-03

**Authors:** Jung-Hwan Baek, Myung Sup Kim, Hye Ryeon Jung, Min-Seon Hwang, Chan-ho Lee, Dai Hoon Han, Yong-ho Lee, Eugene C. Yi, Seung-Soon Im, Ilseon Hwang, Kyungeun Kim, Joon-Yong Chung, Kyung-Hee Chun

**Affiliations:** 1 Department of Biochemistry & Molecular Biology, Graduate School of Medical Science, Brain Korea 21 Project, Yonsei University College of Medicine, Seoul, South Korea; 2 Department of Molecular Medicine and Biopharmaceutical Sciences, School of Convergence Science and Technology and College of Medicine or College of Pharmacy, Seoul National University, Seoul, 03080 South Korea; 3 Department of Surgery, Yonsei University College of Medicine, Seoul, South Korea; 4 Department of Internal Medicine, Yonsei University College of Medicine, Seodaemun-gu, Seoul, 03722 South Korea; 5 Department of Physiology, Keimyung University School of Medicine, Daegu, 42601 South Korea; 6 Department of Pathology, Keimyung University School of Medicine, Daegu, South Korea; 7 Department of Pathology, Kangbuk Samsung Hospital, Sungkyunkwan University School of Medicine, Seoul, 03181 South Korea; 8 Laboratory of Pathology, Center for Cancer Research, National Cancer Institute, National Institutes of Health, Bethesda, MD 20892 USA

**Keywords:** Metabolic disorders, Deubiquitylating enzymes

## Abstract

Nonalcoholic fatty liver disease (NAFLD) occurs due to the accumulation of fat in the liver, leading to fatal liver diseases such as nonalcoholic steatohepatitis (NASH) and cirrhosis. Elucidation of the molecular mechanisms underlying NAFLD is critical for its prevention and therapy. Here, we observed that deubiquitinase USP15 expression was upregulated in the livers of mice fed a high-fat diet (HFD) and liver biopsies of patients with NAFLD or NASH. USP15 interacts with lipid-accumulating proteins such as FABPs and perilipins to reduce ubiquitination and increase their protein stability. Furthermore, the severity of NAFLD induced by an HFD and NASH induced by a fructose/palmitate/cholesterol/trans-fat (FPC) diet was significantly ameliorated in hepatocyte-specific *USP15* knockout mice. Thus, our findings reveal an unrecognized function of USP15 in the lipid accumulation of livers, which exacerbates NAFLD to NASH by overriding nutrients and inducing inflammation. Therefore, targeting USP15 can be used in the prevention and treatment of NAFLD and NASH.

## Liver disease: finding out how to cut the fat

A protein that tags other proteins for destruction causes the accumulation of fat in the liver in nonalcoholic fatty liver disease (NAFLD). Although NAFLD affects ~25% of the global population and can progress to life-threatening liver disease, the only available treatment is weight loss. Kyung-Hee Chun at Yonsei University College of Medicine, Seoul, South Korea, and co-workers found high levels of the protein USP15 in liver tissues from mice fed a high-fat diet and patients with NAFLD. Further experimentation showed that USP15 stabilizes lipid accumulation proteins by removing tags that mark them for destruction. Inactivating the gene encoding USP15 in the liver reduced levels of lipid accumulation proteins, and such USP15-deficient mice showed less liver scarring and fat accumulation. Targeting USP15, perhaps for the development of inhibitors, is a promising approach for new treatments for NAFLD.

## Introduction

Nonalcoholic fatty liver disease (NAFLD) is characterized by an excessive fat build-up in the liver without apparent causes, such as alcohol consumption. NAFLD is the most common liver disease, affecting ~25% of the global population^1–3^. Although the clear causes of NAFLD remain unknown, prolonged NAFLD shows a propensity to progress to nonalcoholic steatohepatitis (NASH), which is characterized by severe lipid accumulation, inflammation, and fibrosis^4,5^. Irregular lipid accumulation and metabolism in the liver further promote insulin resistance and inflammation, exacerbating hepatic steatosis, resulting in a dangerous cycle that is difficult to break. Moreover, NASH may eventually develop into conditions such as type 2 diabetes, cirrhosis, or liver cancer^6,7^. However, the only effective treatment known for NAFLD is weight loss^8^. Therefore, the development of targeted drugs by elucidating the molecular mechanisms underlying NAFLD is important.

Different ubiquitin modifications of proteins elicit specific responses depending on the ubiquitinated substrates. In addition, ubiquitinated substrates can act as novel signaling molecules^9–11^. Conjugation of ubiquitin to substrate proteins leads to proteasomal degradation and can be reversed by deubiquitinating enzymes^12^. The cellular deubiquitination system is involved in various physiological processes^13^. Therefore, the balance between ubiquitination and deubiquitination is strictly regulated. Importantly, dysregulation of protein homeostasis by the ubiquitin system is firmly associated with the pathogenesis of various diseases, including NAFLD and NASH^14–17^. We screened the expression levels of deubiquitinases in liver tissues of mice with NAFLD and focused on USP15 (Supplementary Fig. 1). USP15 is a deubiquitinating enzyme that removes polyubiquitin chains from target proteins. This enzyme is known to stabilize the TGF-β receptor and downstream transducer receptor-regulated SMADs, thus exhibiting an oncogenic nature^18^. In addition, USP15 increases TNF-α− or IL-Iβ-induced NF-κB activity, which results in enhanced inflammatory responses by stabilizing the TGF-β activated kinase 1 binding proteins TAB2/3^19^. USP15 is also known to stabilize MDM2, which is a ubiquitin ligase that causes proteasomal degradation of the tumor suppressor p53 and is associated with reduced tumor cell apoptosis and antitumor T-cell response^20,21^. Recently, USP15 was shown to regulate hepatitis C viral RNA translation and lipid droplet formation in hepatocytes^22^. The extensive impact of USP15 further indicates the possible role for USP15 in the pathogenesis of NAFLD.

Here, we show that the expression of USP15 in the liver is markedly upregulated in individuals with NAFLD and NASH. Furthermore, to evaluate hepatic lipid accumulation and related complications, we selected murine diets known to induce NAFLD by a high-fat diet (HFD) or NASH by a diet rich in fructose, palmitate, and cholesterol (FPC)^23,24^. Our data reveal a molecular mechanism of USP15 in the pathogenesis of NAFLD and NASH phenotypes using USP15 liver-specific knockout mice.

## Materials and methods

### Cell culture and RNAi transfection

AML12 cells were kindly provided by Professor Jae-woo Kim (Yonsei University). Human embryonic kidney 293 (HEK293) cells were purchased from the Korea Cell Line Bank (Seoul, Korea). These cells were maintained in Dulbecco’s modified Eagle’s medium (DMEM) with 10% fetal bovine serum and 1% penicillin/streptomycin at 37 °C in 5% CO_2_. AML12 and HEK293 cells were transfected using Lipofectamine 2000 or Lipofectamine RNAiMax according to the manufacturer’s instructions^25^ (Invitrogen, Carlsbad, CA, USA).

### RNA isolation and quantitative reverse transcription-PCR (qRT‒PCR)

Total RNA was isolated using an RNA lysis reagent (Intron Biotechnology, Korea) as described in a previous study^26,27^. The complementary DNA was synthesized using the quantitative PCR master mix (TOYOBO, Osaka, Japan). qRT‒PCR was performed using SYBR Premix Ex Taq (Clontech Laboratories, Mountain View, CA, USA) on ABI instruments (Applied Biosystems, Inc., Foster City, CA, USA). The expression of genes was normalized to that of β-actin.

### Western blot assay

Cell lysate extractions and tissues were prepared using radioimmunoprecipitation assay (RIPA) buffer (1% Triton X-100; 1% sodium deoxycholate; 0.1% sodium dodecyl sulfate; 150 mM NaCl; 50 mM Tris-HCl, pH 7.5; and 2 mM EDTA, pH 8.0), as described previously^28,29^. Antibodies against USP15, FABP1, FABP4, Perilipin1, Perilipin2, HA, and β-actin were purchased from Santa Cruz Biotechnology (Dallas, TX, USA). The antibody against FLAG was obtained from Sigma‒Aldrich (St. Louis, MO, USA). The normalization control was β-actin.

### Immunoprecipitation

Cell lysate extraction was performed with an immunoprecipitation buffer, as mentioned previously^30^. Cell lysates were immunoprecipitated with antibodies (FLAG, USP15, FABP1, FABP4, Perilipin1, and Perilipin2), and protein A/G agarose beads were added to the lysates, followed by incubation. The immunoprecipitates were washed twice with washing buffer and boiled in SDS sample buffer. After centrifugation, the supernatant was analyzed by Western blotting.

### Ubiquitination assay

Ubiquitination assays were performed as described previously^31^. Briefly, the cells were lysed in phosphate-buffered saline (PBS) containing 5 mM *N*-ethylmaleimide (Sigma-Aldrich) to hinder deubiquitination. The lysate was diluted with 0.9 ml of nondenaturing lysis buffer, followed by fragmentation of the viscous chromatin complexes by the passage of the lysed suspension three to five times through a needle attached to a 1 ml syringe and incubation on ice for 5 min. The dissociated cells were centrifuged at 13,000 rpm for 10 min at 4 °C, followed by immunoprecipitation. For the in vitro deubiquitination assay using TUBE2-agarose pulldown, total ubiquitinated proteins from HEK293 cells were pulled down with 30 μl of TUBE2 beads for 2 h at 4 °C, followed by Western blot analysis^32–34^.

### Oil Red O (ORO) staining

The fatty acid-treated AML12 cells were used as previously described in ref. ^35^. The ORO stock solution (0.35 g/100 ml) was diluted with isopropanol to prepare a 60% ORO working solution. The dried cells were stained with the ORO working solution for 30 min and washed three times with distilled water.

### Mouse experiments

USP15*-*floxed C57BL/6 mice were purchased from the European Mouse Mutant Archive (München, Germany). Liver-specific USP15 knockout mice (*USP15*^*LKO*^) were generated by mating USP15-floxed mice with albumin-Cre mice. Wild-type and *USP15*^*LKO*^ mice were bred as heterozygotes in-house and maintained on a C57BL/6 background. Heterozygous mice were bred with each other to generate wild-type and *USP15*^*LKO*^ mice. Seven-week-old male wild-type and *USP15*^*LKO*^ mice were fed a normal-fat diet (NFD) or an HFD (Research Diets, Inc., New Brunswick, NJ, USA) for 12 weeks. Seven-week-old male wild-type and *USP15*^*LKO*^ mice were fed a fructose-palmitate-cholesterol (FPC) diet (ENVIGO, Indianapolis, IN, USA) for 16 weeks. All mice had free access to food and water and were kept on a 12 h light/12 h dark cycle. Body weight and food intake were measured once a week. The animal studies were approved by the Yonsei University Health System Institutional Animal Care and Use Committee (Permission number for animal experiments: 2015-0073).

### Isolation of murine primary hepatocytes

Isolation of murine primary hepatocytes was performed by standard two-step perfusion protocols with Liberase^36^. Briefly, mice were anesthetized with isoflurane and underwent laparotomy. The liver was perfused with HBSS buffer containing 1 mM EDTA followed by digestion with 0.02 μg/ml Liberase^TM^. The liver was removed and mechanically disassociated and filtered through 70-μm strainers. Then, cells were seeded at a concentration of 8 × 10^6^ cells/dish.

### Histopathology analysis

Mouse livers were fixed with 10% formalin and embedded in paraffin. Paraffin-embedded liver sections were stained with H&E for morphological analysis. Sirius red staining was performed using a Picrosirius Red stain kit according to the manufacturer’s instructions (Abcam, Cambridge, MA, UK). For the terminal deoxynucleotidyl transferase dUTP nick end labeling (TUNEL) assay, paraffin-embedded sections were stained using the DeadEnd^TM^ colorimetric TUNEL system according to the manufacturer’s instructions (Promega Corporation, Madison, WI, USA).

For Oil Red O staining, fresh mouse livers were embedded in OCT solution, and frozen liver sections were fixed in 10% formalin for 10 min and washed with distilled water. Air-dried sections were stained with Oil Red O solution for 15 min and washed with 60% isopropanol. Then, stained sections were washed with distilled water.

### Adenoviruses

The adenovirus expressing murine USP15 was prepared using an AdenoZAP kit (O.D.260, Inc., Boise, Idaho, USA). An adenoviral vector expressing USP15 was recombined with the pZAP1.2 vector according to the manufacturer’s instructions and transfected into HEK293T cells. For the preparation of a crude experimental viral stock from HEK293T cells, the cells were subjected to three freeze/thaw cycles and centrifuged. For adenovirus amplification, HEK293T cells were infected with the viral stock. Large-scale purification was performed with cesium chloride gradient ultracentrifugation according to the standard protocol. For in vivo transduction, 7-week-old male C57BL/6 mice were injected with adenovirus via the tail vein. The doses are described in each figure. All mice were sacrificed seven days after injection.

### Glucose and insulin tolerance test

For the glucose tolerance test (GTT), mice were fasted for 15 h, and 1 g/kg glucose (Sigma-Aldrich) was intraperitoneally injected into mice.

For the insulin tolerance test (ITT), mice were fasted for 6 h, and 1 IU/kg insulin was intraperitoneally injected into the mice. Blood glucose levels were measured 15, 30, 60, 90, and 120 min after injection.

### Mass spectrometry and network analysis

For identification of USP15-interacting proteins in AML12 cells, USP15 immunoprecipitation eluates were separated by SDS‒PAGE and subjected to in-gel digestion and downstream processing as described in our previous report^31^. Extracted peptides were suspended in 0.1% FA in water, loaded onto an EASY-Spray C18 column (75 µm × 50 cm, 2 µm) and separated with a 2–35% gradient of 0.1% FA in ACN for 65 min at a flow rate of 300 nL/min. MS spectra were recorded on a Q-Exactive hybrid quadrupole-Orbitrap mass spectrometer (Thermo Fisher Scientific, San Jose, CA, USA) interfaced with a nanoultra-HPLC system (Easy-nLC1000; Thermo Scientific). Collected MS/MS raw files were converted to mzXML files using the Trans-Proteomic Pipeline (version 4.4) and analyzed using the Sequest (version 27) algorithm in the SORCERER (Sage-N Research, Milpitas, CA, USA) platform. A protein database search was performed using the UniProt human database (version 2016.06, 313072 entries). Full tryptic specificity and up to two missed cleavage sites were allowed. Mass tolerances for precursor ions and fragment ions were set to 10 ppm and 1 Da, respectively. Fixed modification for carbamidomethyl-cysteine (+57.0215 Da) and variable modifications for methionine oxidation (+15.9949 Da) were used. All proteins with a ProteinProphet probability of ≥95% with a minimum of two peptides and a PeptideProphet probability of ≥90% and peptide FDR ≤0.3% were identified using Scaffold (version 4.3.2; Proteome Software, Portland, OR, USA). Network analysis of USP15-interacting proteins was performed by using Ingenuity Pathway Analysis (IPA) software (Ingenuity System, Inc., USA). Protein interaction networks functionally associated with USP15 were merged to generate a protein interaction network.

### Immunohistochemical staining

Sixty-two human liver samples were obtained from individuals who had undergone liver biopsy or transplantation. NAFLD (*n* = 18) and NASH (*n* = 28) were diagnosed by pathologists according to the criteria of Kleiner et al. ^37^. Samples with a NASH activity score (NAS) of 0 were classified as the healthy normal liver. Samples with a NAS of 1–2, a ballooning score of 0 and no fibrosis were classified as NAFLD. Samples with NAS ≥5 or NAS of 3–4 but showing fibrosis were classified as NASH. All procedures were conducted according to the ethical guidelines of the Declaration of Helsinki, and all individuals gave informed consent. The study protocol was approved by the Institutional Review Board at Severance Hospital (IRB No 4–2014–0674, Seoul, South Korea). Immunohistochemistry was performed as previously described in ref. ^38^. Immunohistochemistry was performed on 5 µm formalin-fixed paraffin-embedded tissues. The tissue section was deparaffinized, rehydrated through graded alcohols, and subjected to heat-induced antigen retrieval using antigen retrieval buffer of pH 6.0 (for USP15 & F4/80) or pH 9.0 (for Perilipin1) (Dako, Carpinteria, CA). The endogenous peroxidase activity was quenched with 3% H_2_O_2_ for 10 min. Subsequently, nonspecific binding was blocked with protein block (Dako) for 20 min at room temperature. The sections were incubated with anti-USP15 rabbit polyclonal antibodies (Abcam cat. ab97533) at a 1:500 dilution for 1 h, anti-Perilipin1 rabbit polyclonal antibodies (Abcam, cat. ab3526) at a 1:1000 dilution for 1 h, anti-FABP4 rabbit polyclonal antibodies (Abcam, cat. ab13979) at a 1:2000 dilution for 0.5 h, and anti-F4/80 rabbit monoclonal antibodies (Cell Signaling, D2S9R clone, cat. #70076) at a 1:500 dilution for 1 h. The antigen-antibody reaction was detected with Dako EnVision+ Dual Link System-HRP (Dako) and DAB+ (3, 3’-diaminobenzidine; Dako). Stained sections were lightly counterstained with hematoxylin. Negative controls, including immunoglobulin G (IgG) and omission of the primary antibody, were concurrently performed. The immunopositivity of USP15 and Perilipin1 was assessed by the proportion of positive hepatocytes (0, 0% positive; 1, ≤10%; 2, >10%).

### Quantification and statistical analysis

We employed unpaired *t-*tests for comparisons between the two groups. The statistical analysis was performed using Prism 5 (GraphPad, La Jolla, CA). For IHC data, statistical analyses were performed using PASW Statistics 18 for Windows (version 18.0; IBM SPSS, Inc., Chicago, IL, USA). Crosstabs, Pearson’s *X*^2^ test, and ANOVA were used as needed. *P* values were considered significant when less than 0.05.

## Results

### USP15 is elevated in the liver tissues of mice fed an HFD and in the liver specimens of NAFLD and NASH patients

**Fig. 1:**
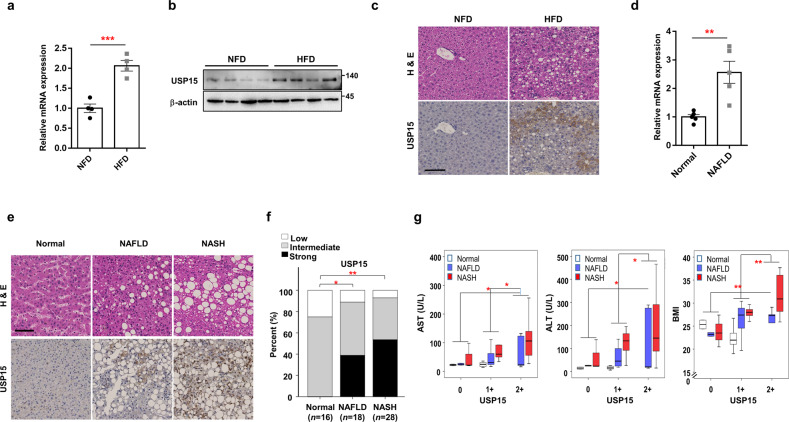
USP15 is upregulated in fatty livers. **a**, **b** The mRNA and protein levels of USP15 in the liver from mice fed a normal-fat diet (NFD) or a high-fat diet (HFD) for 12 weeks (*n* = 4/group). **c** Representative images of mouse normal and NAFLD liver tissues with low and high immunohistochemical staining for USP15. Hepatic steatosis was visualized with hematoxylin and eosin (H&E) staining. Scale bar indicates 100 μm. **d** The level of USP15 mRNA in the livers of individuals without NAFLD (normal) or with NAFLD (*n* = 5/group). **e** Representative immunohistochemical staining for USP15 in formalin-fixed paraffin-embedded liver tissues. H&E staining of formalin-fixed, paraffin-embedded human liver samples diagnosed as normal, NAFLD, and NASH is shown at the top of the image. Scale bar indicates 100 μm. **f** USP15 levels were significantly increased in NAFLD (*p* = 0.041) and NASH (*p* = 0.001) specimens compared to those in normal controls. **g** Positive correlation between USP15 levels and serum AST concentrations and serum ALT concentrations and BMI. *p < 0.05, **p < 0.01, and ***p < 0.001 compared to the NFD or normal group.

The mRNA expression levels of USPs were screened using the liver tissues of mice fed an HFD or NFD for 12 weeks (Supplementary Fig. 1). The levels of most USPs out of 49 deubiquitinases decreased in fatty livers compared to those in normal livers. Interestingly, the mRNA expression of USP50, USP15, and USP39 increased in the fat-accumulated livers. We also reconfirmed the increase in mRNA and protein levels (Fig. 1a, b) of USP15 in the liver tissues of the HFD-fed mice. IHC analysis revealed that USP15 expression was increased in the cytosol of hepatocytes in the HFD-fed mice (Fig. 1c).

The expression of USP15 was also detected in the liver tissues of NAFLD and NASH patients. The mRNA levels of USP15 were increased (Fig. 1d), and a gradient increment of USP15 proteins was detected in the hepatocytes in the NAFLD and NASH patients (Fig. 1e). The percentage of strong expression of USP15 in patients was gradually increased in the patient population with NAFLD and NASH (Fig. 1f). Moreover, the increase in USP15 was parallel to the levels of AST and ALT and BMI scores in the NAFLD and NASH stages (Fig. 1g).

### USP15 directly interacts with lipid accumulation-associated proteins

**Fig. 2:**
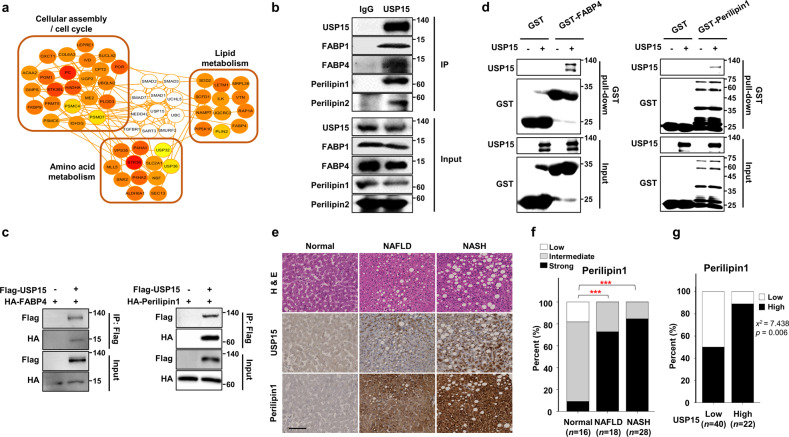
USP15 interacts with FABP1 and FABP4 and Perilipin1 and Perilipin2. **a** USP15 interactome analysis. USP15 binding proteins were immunoprecipitated using a FLAG antibody and identified via mass spectrometry. **b** Interaction between endogenous USP15 and FABP1, FABP4, Perilipin1, and Perilipin2. The AML12 cells were immunoprecipitated using IgG and USP15 antibodies. **c** The interaction between exogenous USP15 and FABP4 or Perilipin1. HEK293 cells were transfected with FLAG-USP15, HA-FABP4, or HA-Perilipin1 plasmids and immunoprecipitated using a FLAG antibody. **d** GST pulldown assay showing a direct interaction between USP15 and FABP4 or Perilipin1. **e** Representative immunohistochemical staining for USP15 and Perilipin1 in patients with nonalcoholic fatty liver disease. The scale bar indicates 100 µm. **f** Overexpression of *Perilipin1* was observed in NAFLD (*p* < 0.001) and NASH (*p* < 0.001). **g** There was a statistically significant correlation between USP15 and Perilipin1 in nonalcoholic fatty liver specimens (Pearson’s *X*^2^ test = 7.483, *p* = 0.006). ***p < 0.001 compared to normal.

Mass spectrometry was performed to screen USP15 substrates using AML12 mouse hepatocytes (Fig. 2a). Interestingly, we found that USP15 interacts with lipid metabolism-related proteins such as fatty acid-binding protein, FABP4 and Perilipin2. We confirmed the interaction between USP15 and not only FABP4 and Perilipin2 but also FABP1 and Perilipin1, which are expressed in hepatocytes, by immunoprecipitation assays (Fig. 2b, c). We also performed an in vitro GST pulldown assay to detect direct interactions between USP15 and FABP4 and Perilipin1 (Fig. 2d). The mRNA expression of FABP4 and Perilipin1 was also significantly upregulated in the NAFLD patients (Supplementary Fig. 2). Furthermore, increases in FABP4 and Perilipin1 were found in the steatosis and NASH stages using public database (GSE89632 and GSE48452) analysis (Supplementary Fig. 3), suggesting that the regulation of FABPs and perilipins by USP15 is important in NAFLD development.

Next, we determined the immunopositivity of USP15 and Perilipin1 in the liver biopsies of patients with NAFLD and NASH and normal liver tissues. USP15 was stained in the cytoplasm of hepatocytes with a granular pattern and was expressed more strongly in hepatocytes without fat droplets. Perilipin1 also showed cytoplasmic expression but was concentrated around fat droplets (Fig. 2e). A parallel trend was observed for the expression levels of USP15/Perilipin1 and NAFLD disease progression (Fig. 2f). Furthermore, there was a statistically significant correlation between USP15 and Perilipin1 expression in human liver tissues (Pearson’s *X*^2^ test = 7.438, *p* = 0.006, Fig. 2g). The expression of Perilipin1 had a parallel correlation with the worsening of liver function, such as the AST and ALT levels, and with the BMI score (Supplementary Fig. 4). Unfortunately, we could not detect the expression of FABP4 using immunohistochemical assays.

### USP15 increases the protein stability of FABPs and perilipins and lipid accumulation in hepatocytes

**Fig. 3:**
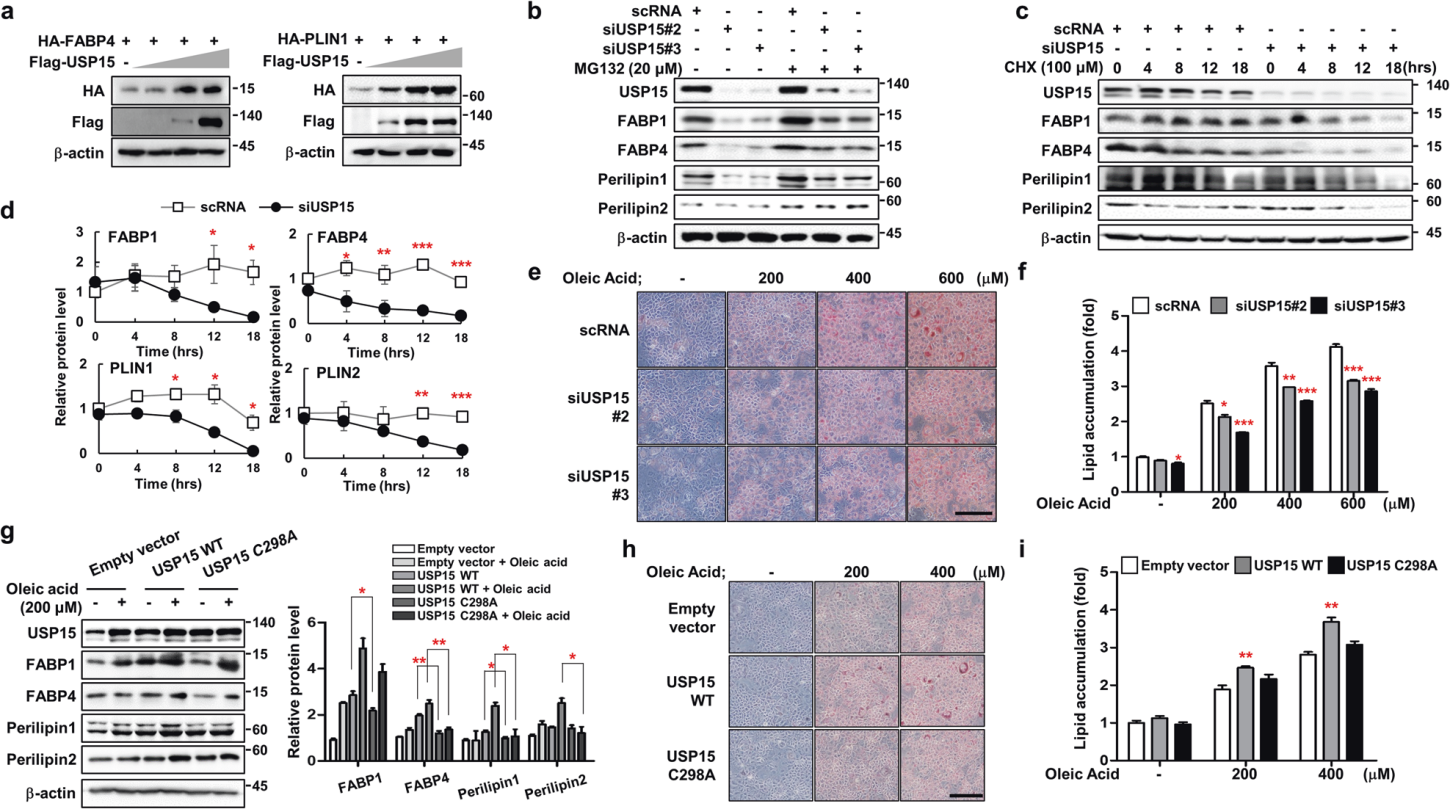
USP15 increases the protein stability of FABP1/4 and Perilipin1/2 and lipid accumulation in hepatocytes. **a** Western blot analysis showing the dose dependency of HA-FABP4 or HA-PLIN1 in HEK293 cells with increasing FLAG-USP15. **b** Effects of USP15 knockdown on the protein stability of FABP1/4 and Perilipin1/2. AML12 cells were transfected with siRNA against USP15, and cells were treated with or without 20 μM MG132 for 8 h. **c** Western blots of AML12 cells treated with cycloheximide as indicated. **d** Normalized protein levels of FABP1/4 and Perilipin1/2 from (**c**). **e** Representative image of Oil Red O staining of AML12 cells treated with oleic acid as indicated with or without siRNA against USP15. **f** Normalization of Oil Red O staining from (**e**). Data were represented as the mean ± SEM. *p < 0.05, **p < 0.01, and ***p < 0.001 compared to scRNA. **g** Western blots of AML12 cells transfected with either USP15 WT or C298A followed by oleic acid treatments as indicated. Data were represented as the mean ± SEM. *p < 0.05 and **p < 0.01 compared to USP15 WT. **h** Representative image of Oil Red O staining of AML12 cells transfected with either USP15 WT or C298A mutant. Cells were treated with oleic acid as indicated. **i** Normalization of Oil Red O staining from (**h**). Data were represented as the mean ± SEM. *p < 0.05 and **p < 0.01 compared to empty vector.

An increase in USP15 increased the level of FABP4 or Perilipin1 in a dose-dependent manner (Fig. 3a and Supplementary Fig. 5). USP15-dependent degradation was mediated by the 26S proteasome complex. MG132, a proteasome inhibitor, inhibited the protein degradation of FABP1, FABP4, Perilipin1, and Perilipin2 when USP15 was downregulated by siRNA targeting USP15 (Fig. 3b). To determine whether the decreased protein levels of FABPs and perilipins due to interference of USP15 expression with siRNA resulted from the decreased protein half-life, we treated the cells with cycloheximide, a protein synthesis inhibitor. The AML12 cells transfected with siRNA targeting USP15 showed a significantly reduced protein half-life of FABPs and perilipins (Fig. 3c, d).

Furthermore, we elucidated the function of USP15 in lipid accumulation through the stabilization of these proteins. We treated the cells with oleic acid and performed ORO staining in AML12 cells. The results showed that the oleic acid-treated cells accumulated a substantial amount of lipids in a dose-dependent manner, whereas the cells transfected with USP15 siRNA showed reduced lipid accumulation (Fig. 3e, f). We also confirmed that palmitic acid treatment induced a significant increase in the protein levels of USP15 in mouse hepatocytes (Supplementary Fig. 6).

Overexpression of wild-type USP15 (USP15 WT) further increased the protein levels of FABPs and perilipins, whereas USP15 C298A, a dominant-negative form of USP15, did not result in protein increases (Fig. 3g). Consistent with previous data, ORO staining indicated that overexpression of USP15 WT resulted in an increase in lipid accumulation in a dose-dependent manner, whereas USP15 C298A failed to result in lipid accumulation (Fig. 3h, i). Thus, USP15 plays a critical role in mediating the protein stability of FABPs and perilipins, which results in lipid accumulation.

### USP15 regulates the protein stability of FABPs and perilipins via deubiquitination

**Fig. 4:**
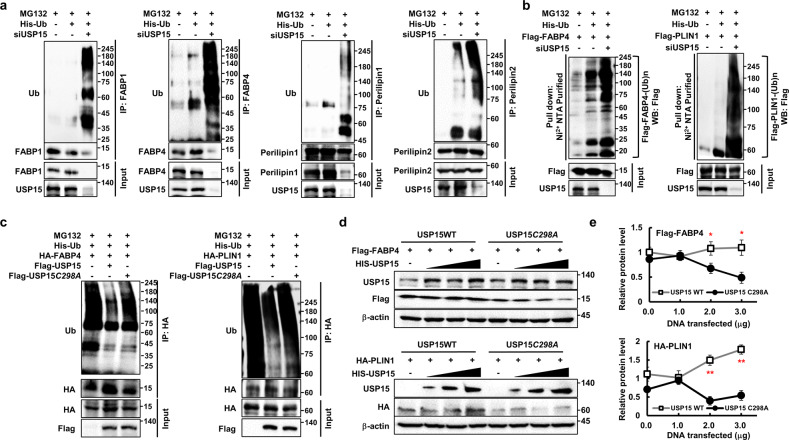
USP15 regulates the protein stability of FABP1/4 and Perilipin1/2 by deubiquitination. **a** Western blots of deubiquitination assays of FABP1/4 and Perilipin1/2. AML12 cells were transfected with or without siRNA against USP15 followed by 20 μM MG132 for 8 h. **b** Western blots of Ni^2+^ NTA pulldown show the deubiquitination of overexpressed *FABP4* or *Perilipin1* with 20 μM MG132 for 8 h. **c** Western blots of deubiquitination assays in HEK293 cells after transfection with either USP15 WT or C298A mutant followed by 20 μM MG132 for 8 h. **d** Effects of USP15 WT or C298A mutant on the protein stability of FABP4 or Perilipin1 in a USP15 dose-dependent manner. **e** Normalization of Western blots of protein levels in (**d**). Data were represented as the mean ± SEM. *p < 0.05, **p < 0.01 com*p*ared to USP15 WT.

We reasoned that USP15 could regulate protein stability by affecting the ubiquitination status of FABPs and perilipins. We found that the polyubiquitination of FABPs and perilipins increased in response to the elimination of USP15 in AML12 cells (Fig. 4a and Supplementary Fig. 7) and murine primary hepatocytes (Supplementary Fig. 8). In addition, Ni^2+^ NTA pulldown further confirmed that USP15 is indeed the deubiquitination enzyme for FABP4 and Perilipin1 (Fig. 4b). To further confirm the deubiquitination of FABPs and perilipins mediated by USP15, we used TUBE-agarose beads (tandem ubiquitin-binding entities) to identify and isolate total ubiquitinated proteins. Consistent with previous findings, knockdown of USP15 by siRNA resulted in increased deubiquitination of FABP4 and Perilipin2 (Supplementary Fig. 9). Overexpression of USP15 WT in HEK293 cells caused a reduction in either FABP4 or Perilipin1 ubiquitination, whereas USP15 C298A did not reduce the ubiquitination of FABP4 and Perilipin1 (Fig. 4c). In concert with the USP15-mediated deubiquitination assays, the overexpression of USP15 WT significantly increased the total protein levels of FABP4 or Perilipin1, whereas USP15 C298A exhibited a severe reduction in the protein levels (Fig. 4d, e and Supplementary Fig. 10). Together, our results indicate that the protein stability of FABPs and perilipins is regulated by USP15-mediated deubiquitination.

### Reduction in the NAFLD phenotype induced by PPARγ-adenovirus in liver-specific USP15 knockout mice

To confirm the function of USP15 in NAFLD, we generated liver-specific USP15 knockout (*USP15*^*LKO*^) mice (Supplementary Fig. 11a). The USP15 protein level was decreased in the liver from the *USP15*^*LKO*^ mice compared with that of the WT mice, whereas no significant difference was observed in the adipose tissues from the WT or *USP15*^*LKO*^ mice (Supplementary Fig. 11b). Body weight, liver weight, liver weight to body weight ratio (Supplementary Fig. 12a), and liver phenotype (Supplementary Fig. 12b) were not different between the WT and *USP15*^*LKO*^ mice fed the NFD. In addition, there was no difference in the serum levels of glucose, triglyceride, free-fatty acid (FFA), cholesterol, AST, and ALT between the WT and *USP15*^*LKO*^ mice under an NFD (Supplementary Fig. 12c).

**Fig. 5:**
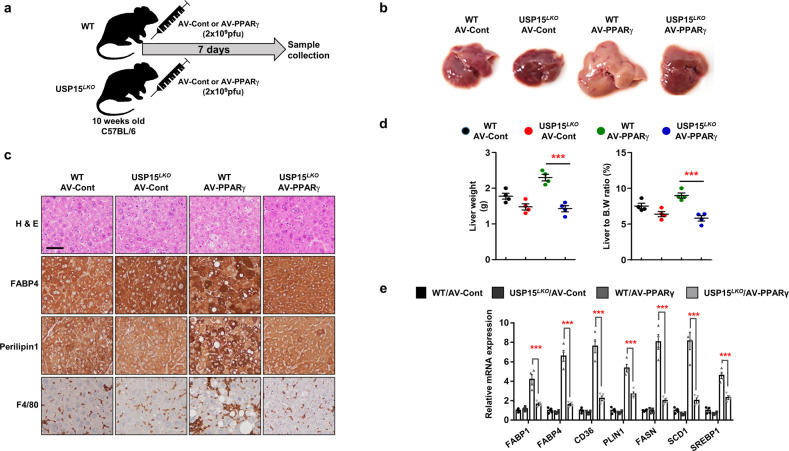
Liver-specific USP15 knockout mice exhibit improved hepatic steatosis in PPARγ-overexpressing livers. **a** Schematic representation of adenovirus injection (*n* = 4/group). **b** Macroscopic view of the liver from adenovirus-injected mice. **c** Slide sections of the liver from adenovirus-injected mice. Liver sections were stained with H&E, FABP4, Perilipin1, and F4/80. **d** Liver weight and the ratio of liver weight to body weight of adenovirus-injected mice (*n* = 4/group). **e** Expression of genes involved in fatty acid uptake and lipogenesis (*n* = 4/group). Data in (**d**, **e**) are represented as the mean ± SEM. ***p < 0.001 compared to WT AV-PPARγ.

Dietary, genetic, and chemically induced mouse models are commonly used as NAFLD animal models^39^. We used a method to induce NAFLD in a short period of time by overexpressing a specific gene using an adenovirus-mediated expression system^40,41^. We searched the literature to find possible upstream regulators of FABPs and perilipins, as well as targets for USP15 that induce NAFLD when overexpressed in the liver. PPARγ is known to upregulate the expression of genes involved in fatty acid uptake and lipid accumulation, including FABP4 and Perilipin1. Adenovirus-mediated PPARγ overexpression induced NAFLD in mice^42^. We injected PPARγ-expressing adenovirus into WT or *USP15*^*LKO*^ mice via the tail vein (Fig. 5a). PPARγ-mediated hepatic lipid accumulation was significantly alleviated in the *USP15*^*LKO*^ mice compared to the WT mice (Fig. 5b, c). The increase in FABP4 and perilipin1 expression and macrophage infiltration, as markers of inflammation, was also alleviated in the *USP15*^*LKO*^ mice (Fig. 5c). Liver weight and liver weight to body weight ratio were also reduced in the *USP15*^*LKO*^ mice (Fig. 5d). Genes related to fatty acid accumulation, such as FABPs, perilipins, and CD36, and lipogeneses, such as FASN, SCD-1, SREBP, and PPARγ, were significantly downregulated in the *USP15*^*LKO*^ mice (Fig. 5e).

### The NAFLD phenotype induced by PPARγ-adenovirus was further increased by coadministration with USP15 overexpressing-adenovirus

**Fig. 6:**
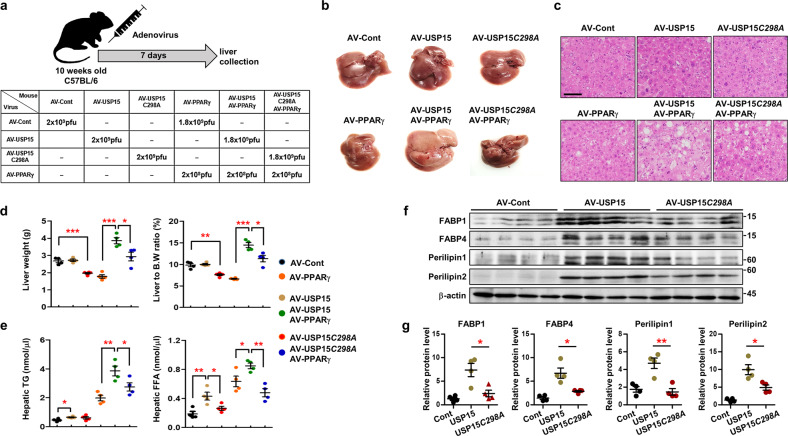
Adenovirus-mediated USP15 overexpression aggravates hepatic steatosis. **a** Schematic representation of adenovirus injection (*n* = 4/group). **b** Macroscopic view of the liver from adenovirus-injected mice. **c** Slide sections of the liver from adenovirus-injected mice. The liver sections were stained with H&E. **d** The liver weight and the ratio of liver weight to body weight of adenovirus-injected mice (*n* = 4/group). Data were represented as the mean ± SEM. *p < 0.05, **p < 0.01, and ***p < 0.001 compared to AV-Cont or AV-PPAR**γ** + USP15. **e** Contents of triglycerides and free fatty acids in the liver from adenovirus-injected mice (*n* = 4/group). Data were represented as the mean ± SEM. *p < 0.05, **p < 0.01, and ***p < 0.001 compared to AV-Cont or AV-PPAR**γ** + USP15. **f** The in vivo effects of USP15 WT or C298A mutant on FABP1/4 and Perilipin1/2 protein stability from the mouse liver (*n* = 4/group). **g** Normalization of protein levels from (d). Data are represented as the mean ± SEM. *p < 0.05 and **p < 0.01 compared to AV-USP15.

We further tested the effect of USP15 overexpression on NAFLD development. WT or C298A mutant USP15-expressing adenoviruses were injected into the tail vein of WT mice with or without PPARγ adenovirus (Fig. 6a). In the absence of PPARγ-adenovirus, the fatty liver phenotype was not observed in the livers of mice injected with USP15*-*adenovirus or USP15 C298A-adenovirus (Fig. 6b, c), whereas the liver weight and liver to body weight ratio of mice injected with USP15 C298A-adenovirus were slightly reduced (Fig. 6d). In the presence of PPARγ-adenovirus, the hepatic steatosis effect of PPARγ-adenovirus was dramatically enhanced by USP15 WT-adenovirus administration (Fig. 6b–d). Interestingly, the prominent hepatic steatosis effect of the PPARγ-adenovirus was less potentiated by the administration of USP15 C298A-adenovirus (Fig. 6b–d), suggesting that the overexpression of USP15 WT in the liver aggravated hepatic steatosis compared to overexpression of USP15 C298A. The concentrations of triglycerides and free fatty acids were highest in liver tissues injected with USP15 WT along with PPARγ-adenovirus (Fig. 6e). We also confirmed that administration of USP15 WT-adenovirus led to a significant increase in the protein levels of FABPs and perilipins in liver tissues, whereas the increase in USP15 C298A-adenovirus-infected livers was less than that of USP15 WT (Fig. 6f, g).

### HFD-induced NAFLD phenotypes were ameliorated in *USP15*^*LKO*^ mice with less inflammation

After 12 weeks of HFD administration, no significant differences in body weight were observed between WT and *USP15*^*LKO*^ mice (Supplementary Fig. 13a,b). We measured metabolic parameters such as food intake, drinking, oxygen consumption, energy expenditure, and physical activity in the WT and *USP15*^*LKO*^ mice (Supplementary Figs. 13c, 14). Energy expenditure in the WT mice was slightly higher in the dark cycle (Supplementary Fig. 14), but other metabolic parameters did not differ between the WT and *USP15*^*LKO*^ mice. The adipose tissue weight and adipose tissue weight to body weight ratio of the WT mice were similar to those of the *USP15*^*LKO*^ mice (Supplementary Fig. 15).

**Fig. 7:**
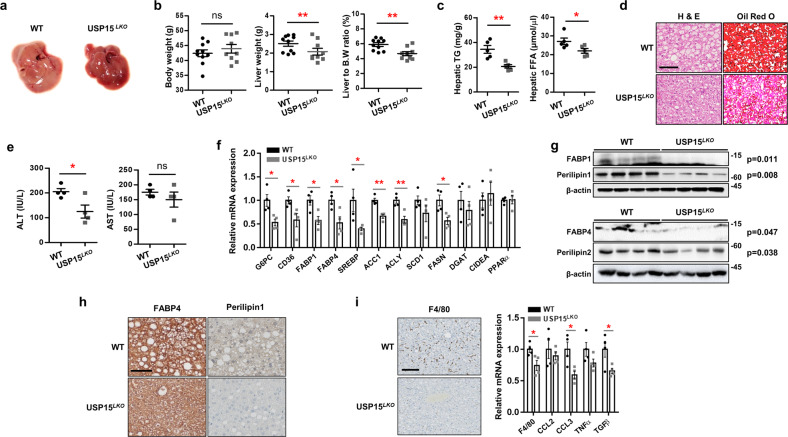
Liver-specific USP15 knockout mice show high-fat diet-induced fatty liver improvement. Both wild-type and *USP15*^*LKO*^ mice were fed a high-fat diet (HFD) for 12 weeks. **a** Macroscopic view of livers from the wild-type and *USP15*^*LKO*^ mice fed an HFD. **b** Body weight, liver weight, and the ratio of liver weight to body weight of the wild-type and *USP15*^*LKO*^ mice fed an HFD (WT: *n* = 10, *USP15*^*LKO*^: *n* = 9). **c** Triglyceride and free fatty acid contents in the livers of the wild-type and *USP15*^*LKO*^ mice fed an HFD (*n* = 5/group). **d** Slide sections of livers from the wild-type and *USP15*^*LKO*^ mice fed an HFD. Liver sections were stained with H&E or Oil Red O. Scale bar indicates 100 μm. **e** Concentrations of ALT and AST in the serum from the wild-type and *USP15*^*LKO*^ mice fed an HFD (*n* = 4/group). **f** The expression of genes involved in gluconeogenesis, fatty acid uptake, lipogenesis, and fatty acid oxidation in the livers of the wild-type and *USP15*^*LKO*^ mice fed an HFD (*n* = 4/group). **g** Protein expression of FABP1, FABP4, Perilipin1, and Perilipin2 in the liver from the wild-type and *USP15*^*LKO*^ mice fed an HFD. **h** Slide sections of livers from the wild-type and *USP15*^*LKO*^ mice fed an HFD. Liver sections were immunohistochemically stained using antibodies against FABP4 and PLIN1. The scale bar indicates 100 μm. **i** The expression of factors involved in inflammation in the liver from the wild-type and *USP15*^*LKO*^ mice fed an HFD (*n* = 4/group). Slide sections of the liver were stained using an antibody against F4/80. The scale bar indicates 100 μm. The mRNA expression was analyzed with quantitative RT‒PCR. Data were represented as the mean ± SEM. *p < 0.05 and **p < 0.01 compared to WT.

However, the *USP15*^*LKO*^ mice exhibited less hepatic steatosis (Fig. 7a) and a lower liver weight and liver to body weight ratio than the WT mice (Fig. 7b). The contents of triglycerides and free fatty acids in the liver were also significantly reduced in the *USP15*^*LKO*^ mice (Fig. 7c, d). The ablation of USP15 in the liver led to a marked decrease in serum ALT concentration (Fig. 7e). There was significant downregulation of the expression of genes related to gluconeogenesis, fatty acid uptake, and lipogenesis in the *USP15*^*LKO*^ mice compared to the WT mice (Fig. 7f). The protein levels of FABPs and perilipins were also significantly reduced in the livers of the *USP15*^*LKO*^ mice (Fig. 7g, h). We also found that the *USP15*^*LKO*^ mice had lower expression of factors involved in inflammation (Fig. 7i). Nevertheless, there were no changes in the serum concentrations of glucose, triglyceride, FFA, and cholesterol (Supplementary Fig. 16). The tolerance tests for glucose (GTT) and insulin (ITT) were performed to measure glucose homeostasis, and no difference was found in the glucose clearance and insulin sensitivity between the WT and *USP15*^*LKO*^ mice (Supplementary Fig. 17).

### Hepatocyte deletion of USP15 suppresses liver steatosis, inflammation, and fibrosis in mouse models of NASH

**Fig. 8:**
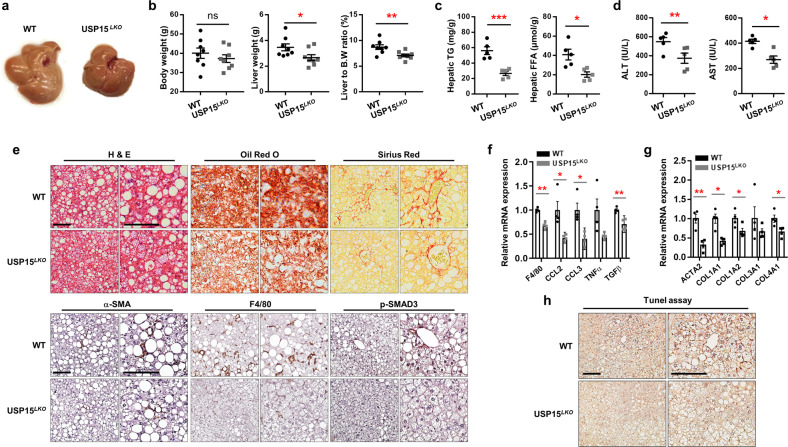
Hepatocyte deletion of USP15 reduces liver steatosis, inflammation, and fibrosis in FPC-fed mice. Both wild-type and *USP15*^*LKO*^ mice were fed a fructose-palmitate-cholesterol (FPC) diet for 16 weeks. **a** Macroscopic view of the liver from the wild-type and *USP15*^*LKO*^ mice fed an FPC diet. **b** Body weight, liver weight, and the ratio of liver weight to body weight of the wild-type and *USP15*^*LKO*^ mice fed an FPC diet (WT: *n* = 8, *USP15*^*LKO*^: *n* = 8). **c** Triglyceride and free fatty acid contents in the livers of the wild-type and *USP15*^*LKO*^ mice fed an FPC diet (*n* = 5/group). **d** Concentrations of ALT and AST in the serum from the wild-type and *USP15*^*LKO*^ mice fed an FPC diet (*n* = 5/group). **e** Slide sections of the liver from the wild-type and *USP15*^*LKO*^ mice fed an FPC diet. Liver sections were stained with H&E, Oil Red O, and Sirius red. Liver sections were immunohistochemically stained using antibodies against α-SMA, F4/80, and p-SMAD3. The left panels represent 200x magnifications (x200), and the right panels represent 400x magnifications (x 400). The scale bar indicates 100 μm. **f** The expression of factors involved in inflammation in the liver from wild-type and *USP15*^*LKO*^ mice fed an FPC diet (*n* = 4/group). The mRNA expression was analyzed with quantitative RT‒PCR. **g** The expression of liver fibrosis markers in the livers from the wild-type and *USP15*^*LKO*^ mice fed an FPC diet (*n* = 4/group). The mRNA expression was analyzed with quantitative RT‒PCR. **h** Terminal deoxynucleotidyl transferase dUTP nick end labeling (TUNEL) assay of liver sections from the wild-type and *USP15*^*LKO*^ mice fed an FPC diet. The scale bar indicates 100 μm. Data were represented as the mean ± SEM. *p < 0.05, **p < 0.01, and ***p < 0.001 compared to WT.

As the HFD induces mild inflammation and fibrosis, we utilized the fructose/palmitate/cholesterol/trans-fat (FPC) diet, which has been shown to induce more severe inflammation and fibrosis^43^. After 16 weeks of FPC diet feeding, the FPC-fed *USP15*^*LKO*^ mice did not show significant differences in body weight and adipose tissue weight compared with the WT mice (Supplementary Fig. 18). However, similar to what was observed in the HFD model, the *USP15*^*LKO*^ mice exhibited less hepatic steatosis (Fig. 8a) and significantly lower liver weight and liver to body weight ratio than the WT controls (Fig. 8b). The *USP15*^*LKO*^ mice also exhibited lower contents of triglycerides and free fatty acids in the liver than the WT mice (Fig. 8c). The ablation of USP15 in the liver led to a marked reduction in serum ALT/AST concentration (Fig. 8d), whereas serum concentrations of triglyceride, free fatty acid, and total cholesterol only showed marginal differences (Supplementary Fig. 19). Notably, lipid accumulation and collagen deposition in the liver were significantly decreased in the livers of the *USP15*^*LKO*^ mice, as confirmed by Oil Red O staining and Sirius Red staining, respectively (Fig. 8e). Additionally, the expression of a profibrotic marker, alpha smooth muscle actin, or inflammation marker, F4/80, was reduced in the *USP15*^*LKO*^ mice compared to the WT controls. Of note, Smad3 is known to promote liver fibrosis by directly binding to the promoter regions of collagens upon phosphorylation^44–46^. The *USP15*^*LKO*^ mice showed much less intense phosphorylation of SMAD3 in the nucleus (Fig. 8e). We also observed reduced FABP4 and Perilipin1 expression in the livers of the mice fed the FPC diet (Supplementary Fig. 20). In addition, there was significant downregulation of gene expression related to inflammation and fibrosis in the *USP15*^*LKO*^ mice in response to the FPC diet (Fig. 8f, g). USP15 deficiency also led to a considerably lower degree of cell death, as confirmed by the TUNEL assay^47^, which labels the free 3’-hydroxyl termini of genomic DNA, indicating apoptotic cell death (Fig. 8h). However, tolerance tests for glucose and insulin did not show either enhanced glucose tolerance or insulin sensitivity due to hepatic USP15 depletion (Supplementary Fig. 21). Therefore, these results strongly suggest that ablation of USP15 improves hepatic steatosis without notable systemic side effects.

## Discussion

To elucidate the molecular mechanisms that govern the pathogenesis of chronic liver diseases, such as NAFLD and NASH, we report here evidence supporting the regulation of protein stability in their development. The degree of ubiquitination and the management of protein homeostasis by the 26S proteasome plays a central role in disease development and progression. In this regard, it is inferred that the regulation of protein homeostasis through deubiquitination is important in the pathogenesis of NAFLD and NASH. Several deubiquitinases were previously shown to be reduced in hepatic steatosis and have a protective function against hepatic steatosis^14,15,17,48^. For example, the expression of USP10 was significantly reduced in mice with NAFLD and obese mice, whereas USP10 overexpression was shown to lower the risk of metabolic dysfunction in mice^15^. In addition, CYLD is known to suppress NASH, reducing lipid accumulation, insulin resistance, inflammation, and fibrosis in mice. However, the interaction of CYLD with the E3 ligase TRIM47 results in the degradation of CYLD in proportion to NASH progression^14^. In this study, we screened the expression levels of deubiquitinases in liver tissues of high-fat diet-fed mice. Interestingly, the expression of USP15 was significantly elevated in the liver tissues of the HFD-fed mice. The expression of USP39 and 50 was also increased in the livers of the HFD-fed mice compared to the livers of the normal mice. However, qPCR analysis of the C_T_ values of USP39 and 50 showed higher C_T_ values, indicating low expression in the livers of the HFD-fed mice. We focused on USP15 because it was difficult to determine the loss of function of USP39 and 50 with low expression using the knockout mouse model. Consistently, the liver specimens of patients with either NAFLD or NASH showed markedly increased expression of USP15. These results suggest that increased expression levels of USP15 play an important role in the pathogenesis of NAFLD and NASH in contrast to the reduced expression of most other deubiquitinases. Consistent with this finding, USP15 is known to mediate TGF-β signaling by stabilizing the type I TGF-β receptor, leading to enhancement of TGF-β signaling^18^ and the NF-κB signaling pathway^19^, which is a critical factor in accelerating fatal liver diseases such as NASH and cirrhosis^49,50^. These results show that USP15 possesses both proinflammatory potential and lipid accumulation ability, suggesting that inhibition of USP15 can be a promising therapeutic approach for NAFLD/NASH.

Here, we determined the direct substrates of USP15 that regulate their stability to support the NAFLD/NASH phenotype. Mass spectrometry analysis revealed that USP15 physically interacts with proteins related to lipid metabolism, fatty acid-binding proteins (FABPs), and perilipins. FABPs are known to be involved in the uptake of long-chain fatty acids and further metabolism. Tissue-specific FABP proteins are differentially expressed. FABP1 is known to be highly expressed in the liver, whereas FABP4 is abundant in adipocytes and macrophages^51^. In previous studies, FABP1 deficiency was reported to result in reduced hepatic triglyceride accumulation and inflammatory marker gene expression in mice fed an HFD^52^. FABP4-deficient mice showed reduced insulin resistance with increased body weight in HFD-induced mice^53^. FABP4 is speculated to be involved in lipolysis, leading to weight gain in FABP4-deficient mice. In addition, perilipins are known to associate with the surface of lipid droplets and control lipid accumulation and inflammation^54^. *PLIN1* is mainly expressed in adipocytes, and its deficiency in HFD-fed mice results in reduced accumulation of triglycerides in white adipose tissues^54^. Perilipin2 is a major hepatic lipid droplet-associated protein; its overexpression leads to considerable lipid droplet accumulation^55^. *PLIN2* knockout mice reportedly show attenuated fatty liver disease and obesity^56^. In our in vivo studies, overexpression of USP15 WT with PPARγ by adenovirus was sufficient to induce stabilization of FABP and PLIN proteins, whereas the catalytically inactive mutant form of USP15 C298A and PPARγ failed to induce protein stabilization in the liver. These data may be direct evidence that the deubiquitinating function of USP15 on FABPs and perilipins could enhance NAFLD/NASH. It is also strongly supposed that FABPs and perilipins are deubiquitinated by USP15 and that their enhanced stability synergistically contributes to increased lipid accumulation and inflammation in the development of NAFLD/NASH.

An FPC diet is used to induce features of metabolic and hepatic NASH characteristics. High sugar and palmitic acid contents are known to promote lipid synthesis and proinflammatory cytokine production, respectively^57^. Additionally, high levels of cholesterol and trans-fat content cause further inflammation and liver damage^58^. In this study, we believe that the absence of USP15 in hepatocytes led to reduced inflammation and fibrosis not only by lowering the expression of FABPs and perilipins but also by suppressing TGF-β signaling pathways and cell death. Interestingly, the USP15 knockout livers of HFD- or FPC diet-fed mice showed not only significantly reduced inflammatory responses and fibrosis but also hepatic triglycerides and free fatty acids. Interestingly, the *USP15*^*LKO*^ mice fed an HFD or FPC diet did not show a significant difference in body weight when compared to the wild-type mice. The fat mass measured in white adipose tissues or glucose metabolism measured with GTT or ITT also did not show any difference. These findings are indicative of the important role played by liver USP15 in the regulation of lipid accumulation and inflammatory responses in only the liver. However, we should not overlook that these phenotypes are exhibited in liver-specific deletion of USP15, not in global deletion of USP15. This method could result in fewer side toxic effects on the whole body, but this issue needs further study.

Taken together, our findings have important implications for targeting deubiquitinases, regulators of protein stability, for NAFLD/NASH treatment. USP15 is involved in the protein stability of various substrates, such as FABP, perilipins, and TGF signaling pathway-associated proteins, thereby governing multiple etiologies of diseases, such as lipid accumulation, inflammatory response, and fibrosis, which are important for NAFLD/NASH therapy. Thus, targeting USP15 by developing specific inhibitors could represent a promising therapeutic strategy.

## Supplementary information

Revised Supplemental Material

## Data Availability

Data supporting the present study are available from the corresponding author upon reasonable request.

## References

[1] Byrne, C. D. & Targher, G. NAFLD: a multisystem disease. *J. Hepatol.* **62**, S47–S64 (2015). 25920090 10.1016/j.jhep.2014.12.012

[2] Danford, C. J. & Lai, M. NAFLD: a multisystem disease that requires a multidisciplinary approach. *Frontline Gastroenterol*. **10**, 328–329 (2019). 31682642 10.1136/flgastro-2019-101235 PMC6788273

[3] Young, S. et al. Prevalence and profile of nonalcoholic fatty liver disease in lean adults: systematic review and meta-analysis. *Hepatol. Commun.* **4**, 953–972 (2020). 32626829 10.1002/hep4.1519 PMC7327210

[4] Michelotti, G. A., Machado, M. V. & Diehl, A. M. NAFLD, NASH and liver cancer. *Nat. Rev. Gastroenterol. Hepatol.* **10**, 656–665 (2013). 24080776 10.1038/nrgastro.2013.183

[5] Eslam, M., Valenti, L. & Romeo, S. Genetics and epigenetics of NAFLD and NASH: Clinical impact. *J. Hepatol.* **68**, 268–279 (2018). 29122391 10.1016/j.jhep.2017.09.003

[6] Rinella, M. E. & Sanyal, A. J. Management of NAFLD: a stage-based approach. *Nat. Rev. Gastroenterol. Hepatol.* **13**, 196–205 (2016). 26907882 10.1038/nrgastro.2016.3

[7] Younes, R. & Bugianesi, E. Should we undertake surveillance for HCC in patients with NAFLD. *J. Hepatol.* **68**, 326–334 (2018). 29122695 10.1016/j.jhep.2017.10.006

[8] Kenneally, S., Sier, J. H. & Moore, J. B. Efficacy of dietary and physical activity intervention in non-alcoholic fatty liver disease: a systematic review. *BMJ Open Gastroenterol*. **4**, e000139 (2017). 28761689 10.1136/bmjgast-2017-000139 PMC5508801

[9] Chen, Z. et al. Ubiquitin-specific protease 1 acts as an oncogene and promotes lenvatinib efficacy in hepatocellular carcinoma by stabilizing c-kit. *Ann. Hepatol.* **27**, 100669 (2022). 35045360 10.1016/j.aohep.2022.100669

[10] Du, Z., Wu, T., Liu, L., Luo, B. & Wei, C. Ubiquitin specific peptidase 1 promotes hepatic fibrosis through positive regulation of CXCL1 by deubiquitinating SNAIL. *Dig. Liver Dis.* **54**, 91–102 (2022). 33926817 10.1016/j.dld.2021.02.025

[11] Kamoshita, K. et al. Insulin suppresses ubiquitination via the deubiquitinating enzyme USP14, independent of proteasome activity in H4IIEC3 hepatocytes. *J. Pharmacol. Exp. Ther*. 10.1124/jpet.122.001088 (2022). 10.1124/jpet.122.001088 36328485

[12] Popovic, D., Vucic, D. & Dikic, I. Ubiquitination in disease pathogenesis and treatment. *Nat. Med.* **20**, 1242–1253 (2014). 25375928 10.1038/nm.3739

[13] Clague, M. J., Urbe, S. & Komander, D. Breaking the chains: deubiquitylating enzyme specificity begets function. *Nat. Rev. Mol. Cell Biol.* **20**, 338–352 (2019). 30733604 10.1038/s41580-019-0099-1

[14] Ji, Y. X. et al. The deubiquitinating enzyme cylindromatosis mitigates nonalcoholic steatohepatitis. *Nat. Med.* **24**, 213–223 (2018). 29291351 10.1038/nm.4461

[15] Luo, P. et al. Ubiquitin-specific peptidase 10 (USP10) inhibits hepatic steatosis, insulin resistance, and inflammation through Sirt6. *Hepatology* **68**, 1786–1803 (2018). 29698567 10.1002/hep.30062

[16] Ghosh, M. et al. Ubiquitin ligase COP1 controls hepatic fat metabolism by targeting ATGL for degradation. *Diabetes* **65**, 3561–3572 (2016). 27658392 10.2337/db16-0506

[17] Zhang, P. et al. The deubiquitinating enzyme TNFAIP3 mediates inactivation of hepatic ASK1 and ameliorates nonalcoholic steatohepatitis. *Nat. Med.* **24**, 84–94 (2018). 29227477 10.1038/nm.4453

[18] Eichhorn, P. J., Rodon, L., Gonzalez-Junca, A., Baselga, J. & Seoane, J. USP15 stabilizes the TGF-beta receptor I and promotes oncogenesis through the activation of the TGF-beta signal in glioblastoma. *Cancer Res.*10.1158/1538-7445.Am2012-Lb-31 (2012). 10.1038/nm.2619 22344298

[19] Zhou, Q. Q. et al. USP15 potentiates NF-kappa B activation by differentially stabilizing TAB2 and TAB3. *FEBS J.* **287**, 3165–3183 (2020). 31903660 10.1111/febs.15202

[20] Villeneuve, N. F. et al. USP15 negatively regulates Nr12 through deubiquitination of Keap1. *Mol. Cell* **51**, 68–79 (2013). 23727018 10.1016/j.molcel.2013.04.022 PMC3732832

[21] Zou, Q. et al. USP15 stabilizes MDM2 to mediate cancer-cell survival and inhibit antitumor T cell responses. *Nat. Immunol.* **15**, 562–570 (2014). 24777531 10.1038/ni.2885 PMC4032322

[22] Kusakabe, S. et al. USP15 participates in hepatitis C virus propagation through regulation of viral RNA translation and lipid droplet formation. *J. Virol*. 10.1128/JVI.01708-18 (2019). 10.1128/JVI.01708-18 PMC6401473 30626683

[23] Cheng, D. et al. MGAT2 inhibitor decreases liver fibrosis and inflammation in murine NASH models and reduces body weight in human adults with obesity. *Cell Metab.* **34**, 1732–1748.e1735 (2022). 36323235 10.1016/j.cmet.2022.10.007

[24] Knorr, J. et al. Interleukin-18 signaling promotes activation of hepatic stellate cells in mouse liver fibrosis. *Hepatology*10.1002/hep.32776 (2022). 10.1002/hep.32776 PMC9984672 36059147

[25] Kim, N. J. et al. A PDE1 inhibitor reduces adipogenesis in mice via regulation of lipolysis and adipogenic cell signaling. *Exp. Mol. Med.* **51**, 5 (2019). 30635550 10.1038/s12276-018-0198-7 PMC6329698

[26] Baek, J. H., Kim, N. J., Song, J. K. & Chun, K. H. Kahweol inhibits lipid accumulation and induces glucose-uptake through activation of AMP-activated protein kinase (AMPK). *BMB Rep.* **50**, 566–571 (2017). 28602160 10.5483/BMBRep.2017.50.11.031 PMC5720470

[27] Hwang, M. S., Baek, J. H., Song, J. K., Lee, I. H. & Chun, K. H. Tschimganidine reduces lipid accumulation through AMPK activation and alleviates high-fat diet-induced metabolic diseases. *BMB Rep.* **56**, 246–251 (2023). 36646438 10.5483/BMBRep.2022-0211 PMC10140487

[28] Baek, J. H., Kim, D. H., Lee, J., Kim, S. J. & Chun, K. H. Galectin-1 accelerates high-fat diet-induced obesity by activation of peroxisome proliferator-activated receptor gamma (PPARγ) in mice. *Cell Death Dis.* **12**, 66 (2021). 33431823 10.1038/s41419-020-03367-z PMC7801586

[29] Lee, J. H. et al. Isocitrate dehydrogenase 2 protects mice from high-fat diet-induced metabolic stress by limiting oxidative damage to the mitochondria from brown adipose tissue. *Exp. Mol. Med.* **52**, 238–252 (2020). 32015410 10.1038/s12276-020-0379-z PMC7062825

[30] Kim, S. J. et al. Galectin-3 increases gastric cancer cell motility by up-regulating fascin-1 expression. *Gastroenterology* **138**, 1035–1045 (2010). 19818782 10.1053/j.gastro.2009.09.061

[31] Cho, Y. et al. Post-translational modification of OCT4 in breast cancer tumorigenesis. *Cell Death Differ.*10.1038/s41418-018-0079-6 (2018). 10.1038/s41418-018-0079-6 PMC6180041 29511337

[32] Nguyen, T. V. et al. Glutamine triggers acetylation-dependent degradation of glutamine synthetase via the thalidomide receptor cereblon. *Mol. Cell* **61**, 809–820 (2016). 26990986 10.1016/j.molcel.2016.02.032 PMC4889030

[33] Nguyen, T. V. et al. p97/VCP promotes degradation of CRBN substrate glutamine synthetase and neosubstrates. *Proc. Natl Acad. Sci. USA* **114**, 3565–3571 (2017). 28320958 10.1073/pnas.1700949114 PMC5389304

[34] Nguyen, T. V. USP15 antagonizes CRL4(CRBN)-mediated ubiquitylation of glutamine synthetase and neosubstrates. *Proc. Natl Acad. Sci. USA*10.1073/pnas.2111391118 (2021). 10.1073/pnas.2111391118 PMC8501880 34583995

[35] Baek, J. H. et al. Galectin-3 activates PPARgamma and supports white adipose tissue formation and high-fat diet-induced obesity. *Endocrinology* **156**, 147–156 (2015). 25343273 10.1210/en.2014-1374

[36] Charni-Natan, M. & Goldstein, I. Protocol for primary mouse hepatocyte isolation. *STAR Protoc.* **1**, 100086 (2020). 33111119 10.1016/j.xpro.2020.100086 PMC7580103

[37] Kleiner, D. E. et al. Design and validation of a histological scoring system for nonalcoholic fatty liver disease. *Hepatology* **41**, 1313–1321 (2005). 15915461 10.1002/hep.20701

[38] Kim, S. J. et al. Activation of nuclear PTEN by inhibition of Notch signaling induces G2/M cell cycle arrest in gastric cancer. *Oncogene* **35**, 251–260 (2016). 25823029 10.1038/onc.2015.80

[39] Oligschlaeger, Y. & Shiri-Sverdlov, R. NAFLD preclinical models: more than a handful, less of a concern? *Biomedicines*10.3390/biomedicines8020028 (2020). 10.3390/biomedicines8020028 PMC7167756 32046285

[40] Chen, J. L., Lu, X. J., Zou, K. L. & Ye, K. Kruppel-like factor 2 promotes liver steatosis through upregulation of CD36. *J. Lipid Res.* **55**, 32–40 (2014). 23861552 10.1194/jlr.M039453 PMC3927469

[41] Kim, J. H., Jung, D. Y., Nagappan, A. & Jung, M. H. Histone H3K9 demethylase JMJD2B induces hepatic steatosis through upregulation of PPARgamma2. *Sci. Rep.* **8**, 13734 (2018). 30214048 10.1038/s41598-018-31953-x PMC6137221

[42] Lee, Y. J. et al. Nuclear receptor PPARgamma-regulated monoacylglycerol O-acyltransferase 1 (MGAT1) expression is responsible for the lipid accumulation in diet-induced hepatic steatosis. *Proc. Natl Acad. Sci. USA* **109**, 13656–13661 (2012). 22869740 10.1073/pnas.1203218109 PMC3427113

[43] Wang, X. et al. Hepatocyte TAZ/WWTR1 promotes inflammation and fibrosis in nonalcoholic steatohepatitis. *Cell Metab.* **24**, 848–862 (2016). 28068223 10.1016/j.cmet.2016.09.016 PMC5226184

[44] Hsieh, Y. C. et al. (Pro)renin receptor knockdown attenuates liver fibrosis through inactivation ERK/TGF-beta 1/SMAD3 pathway. *Cell Mol. Gastroenter.* **12**, 813–838 (2021). 10.1016/j.jcmgh.2021.05.017 PMC8340309 34087453

[45] Ji, J. et al. Apigenin alleviates liver fibrosis by inhibiting hepatic stellate cell activation and autophagy via TGF-beta 1/Smad3 and p38/PPAR alpha pathways. *PPAR Res.* **2021**, 6651839 (2021). 33574836 10.1155/2021/6651839 PMC7861947

[46] Zhong, X. L. et al. SIRT6 protects against liver fibrosis by deacetylation and suppression of SMAD3 in hepatic stellate cells. *Cell Mol. Gastroenter.* **10**, 341–364 (2020). 10.1016/j.jcmgh.2020.04.005 PMC7327931 32305562

[47] Mirzayans, R. & Murray, D. Do TUNEL and other apoptosis assays detect cell death in preclinical studies? *Int. J. Mol. Sci.* **21**, 9090 (2020). 33260475 10.3390/ijms21239090 PMC7730366

[48] An, S. et al. USP18 protects against hepatic steatosis and insulin resistance through its deubiquitinating activity. *Hepatology* **66**, 1866–1884 (2017). 28718215 10.1002/hep.29375

[49] Yang, L. et al. Transforming growth factor beta signaling in hepatocytes participates in steatohepatitis through regulation of cell death and lipid metabolism in mice. *Hepatology* **59**, 483–495 (2014). 23996730 10.1002/hep.26698 PMC3946696

[50] Sunami, Y. et al. Hepatic activation of IKK/NFkappaB signaling induces liver fibrosis via macrophage-mediated chronic inflammation. *Hepatology* **56**, 1117–1128 (2012). 22407857 10.1002/hep.25711

[51] Furuhashi, M., Saitoh, S., Shimamoto, K. & Miura, T. Fatty acid-binding protein 4 (FABP4): pathophysiological insights and potent clinical biomarker of metabolic and cardiovascular diseases. *Clin. Med. Insights Cardiol.* **8**, 23–33 (2014). 25674026 10.4137/CMC.S17067 PMC4315049

[52] Mukai, T., Egawa, M., Takeuchi, T., Yamashita, H. & Kusudo, T. Silencing of FABP1 ameliorates hepatic steatosis, inflammation, and oxidative stress in mice with nonalcoholic fatty liver disease. *FEBS Open Bio* **7**, 1009–1016 (2017). 28680813 10.1002/2211-5463.12240 PMC5494302

[53] Uysal, K. T., Scheja, L., Wiesbrock, S. M., Bonner-Weir, S. & Hotamisligil, G. S. Improved glucose and lipid metabolism in genetically obese mice lacking aP2. *Endocrinology* **141**, 3388–3396 (2000). 10965911 10.1210/endo.141.9.7637

[54] Sohn, J. H. et al. Perilipin 1 (Plin1) deficiency promotes inflammatory responses in lean adipose tissue through lipid dysregulation. *J. Biol. Chem.* **293**, 13974–13988 (2018). 30042231 10.1074/jbc.RA118.003541 PMC6130955

[55] Orlicky, D. J. et al. Perilipin-2 promotes obesity and progressive fatty liver disease in mice through mechanistically distinct hepatocyte and extra-hepatocyte actions. *J. Physiol.* **597**, 1565–1584 (2019). 30536914 10.1113/JP277140 PMC6418763

[56] Najt, C. P. et al. Liver-specific loss of Perilipin 2 alleviates diet-induced hepatic steatosis, inflammation, and fibrosis. *Am. J. Physiol. Gastrointest. Liver Physiol.* **310**, G726–G738 (2016). 26968211 10.1152/ajpgi.00436.2015 PMC4867327

[57] Joshi-Barve, S. et al. Palmitic acid induces production of proinflammatory cytokine interleukin-8 from hepatocytes. *Hepatology* **46**, 823–830 (2007). 17680645 10.1002/hep.21752

[58] Vergnes, L., Phan, J., Strauss, M., Tafuri, S. & Reue, K. Cholesterol and cholate components of an atherogenic diet induce distinct stages of hepatic inflammatory gene expression. *J. Biol. Chem.* **278**, 42774–42784 (2003). 12923166 10.1074/jbc.M306022200

