# Author Correction: Ablation of the deubiquitinase USP15 ameliorates nonalcoholic fatty liver disease and nonalcoholic steatohepatitis

**DOI:** 10.1038/s12276-024-01343-7

**Published:** 2024-10-28

**Authors:** Jung-Hwan Baek, Myung Sup Kim, Hye Ryeon Jung, Min-Seon Hwang, Chan-ho Lee, Dai Hoon Han, Yong-ho Lee, Eugene C. Yi, Seung-Soon Im, Ilseon Hwang, Kyungeun Kim, Joon-Yong Chung, Kyung-Hee Chun

**Affiliations:** 1 Department of Biochemistry & Molecular Biology, Graduate School of Medical Science, Brain Korea 21 Project, Yonsei University College of Medicine, Seoul, South Korea; 2 Department of Molecular Medicine and Biopharmaceutical Sciences, School of Convergence Science and Technology and College of Medicine or College of Pharmacy, Seoul National University, Seoul, 03080 South Korea; 3 Department of Surgery, Yonsei University College of Medicine, Seoul, South Korea; 4 Department of Internal Medicine, Yonsei University College of Medicine, Seodaemun-gu, Seoul, 03722 South Korea; 5 Department of Physiology, Keimyung University School of Medicine, Daegu, 42601 South Korea; 6 Department of Pathology, Keimyung University School of Medicine, Daegu, South Korea; 7 Department of Pathology, Kangbuk Samsung Hospital, Sungkyunkwan University School of Medicine, Seoul, 03181 South Korea; 8 Laboratory of Pathology, Center for Cancer Research, National Cancer Institute, National Institutes of Health, Bethesda, MD 20892 USA

**Keywords:** Metabolic disorders, Deubiquitylating enzymes

Correction to: *Experimental & Molecular Medicine* 10.1038/s12276-023-01036-7, published online 03 July 2023

After online publication of this article, the authors noticed an error in the Acknowledgements section.

The correct statement of this article should have read as below.

“This study was supported by the Bio & Medical Technology Development Program of the National Research Foundation of Korea (NRF) funded by the Korean government (NRF 2021M3A9E4021818 and NRF-2022M3A9G8082639), the NRF grant awarded by the Korean government (NRF-2022R1A2C2007300) and a faculty research grant of Yonsei University College of Medicine (6-2021-0080)”

The authors apologize for any inconvenience caused.

The original article has been corrected.

