# Supplementary material for: Ablation of the deubiquitinase USP15 ameliorates nonalcoholic fatty liver disease and nonalcoholic steatohepatitis
Source: Exp Mol Med. 2023 Jul 3;55(7):1520–30. doi: 10.1038/s12276-023-01036-7 (PMC10394025; doi:10.1038/s12276-023-01036-7)
Supplement: Supplementary file 1 — Revised Supplemental Material [file 12276_2023_1036_MOESM1_ESM.pdf]

Supplemental information

Ablation of the Deubiquitinase USP15 Ameliorates Nonalcoholic Fatty Liver Disease and Nonalcoholic Steatohepatitis

Jung-Hwan Baek, Myung Sup Kim, Hye Ryeon Jung, Min-Seon Hwang, Chan-ho Lee, Dai Hoon Han, Yong-ho Lee, Eugene C Yi, Seung-Soon Im, Ilseon Hwang, Kyungeun Kim, Joon-Yong Chung, and Kyung-Hee Chun

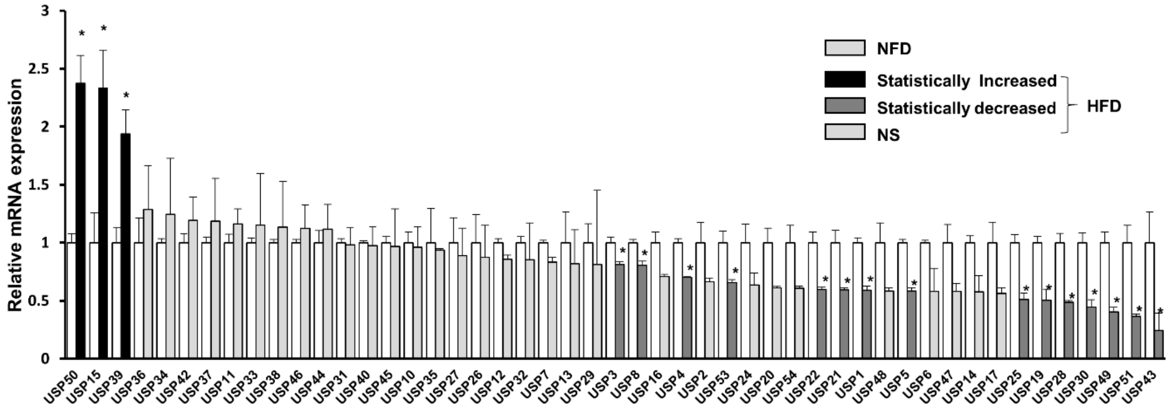

Supplementary Fig. 1. USP15 is upregulated in fatty livers. Mouse liver tissues from NFD or HFD for 12 weeks were used to screen the mRNA expression levels of USPs with statistical significance. USP15, USP39, and USP50 showed marked increase of mRNA expression levels in HFD mouse liver tissues.

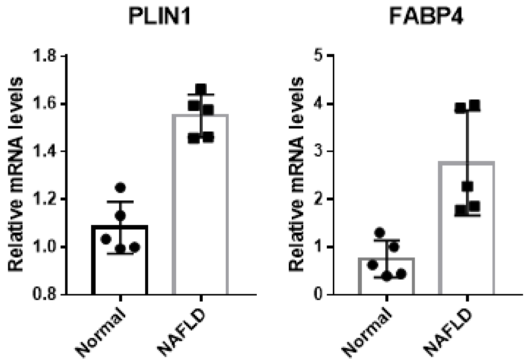

Supplementary Fig. 2. The expression of FABP4 and Perilipin1 is also upregulated in fatty livers. Some of the genes involved in lipid metabolism are also significantly upregulated in the liver tissues of NAFLD patients.

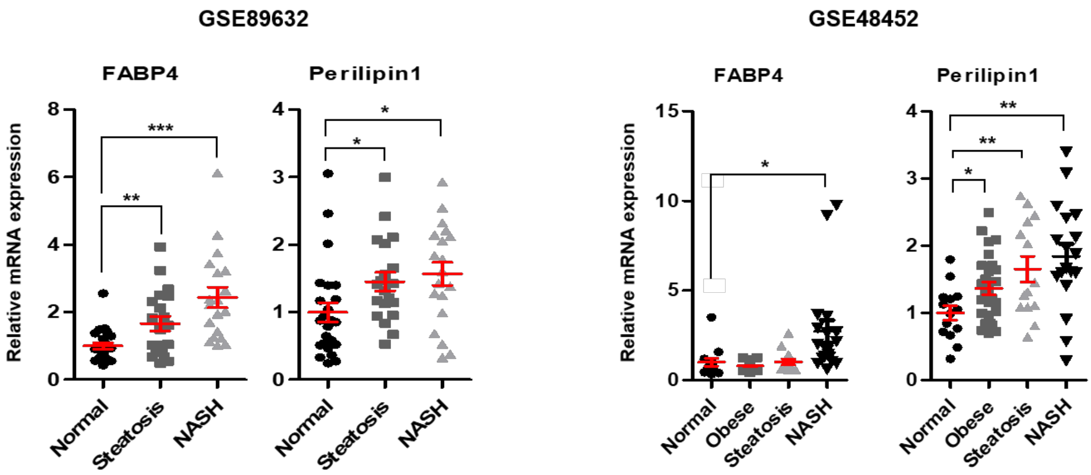

Supplementary Fig. 3. FABP4 and Perilipin1 are upregulated in fatty livers. mRNA expression of FABP4 and Perilipin1 in GSE89632 or GSE48452.

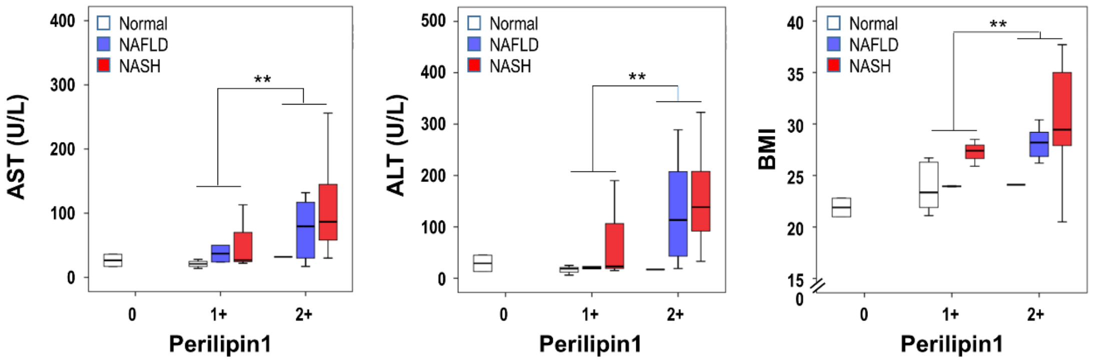

Supplementary Fig. 4. The expression of Perilipin1 is parallel to the levels of AST and ALT and BMI scores in NAFLD and NASH stage. Positive correlation between Perilipin1 level and serum AST concentrations, and serum ALT concentrations and BMI in NAFLD and NASH stage.

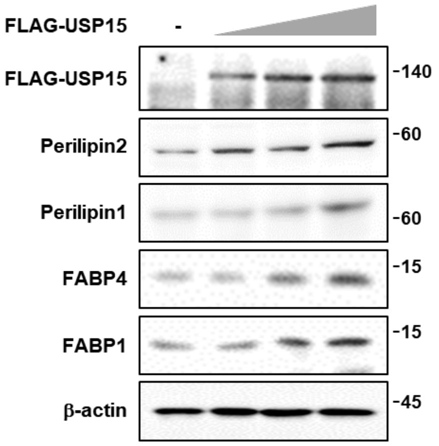

Supplementary Fig. 5. Protein expression of endogenous FABPs and perilipins in response to increment of USP15. Western blot analysis showing dose-dependency of by FLAG-USP15 on endogenous protein levels of FABPs and perilipins in HEK293 cells. Protein expression levels were normalized by β-actin.

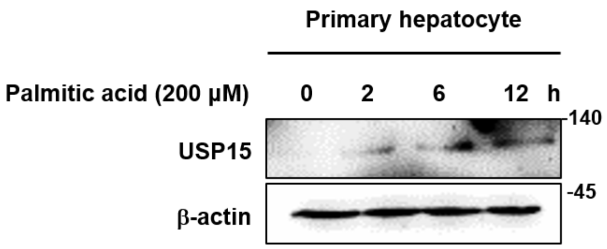

Supplementary Fig. 6. Protein expression of USP15 in mouse primary hepatocyte treated with palmitic acid. Primary hepatocyte isolated from mice stimulated with palmitic acid (200 μM) for up to 12 hrs. Protein expression was normalized to β-actin levels.

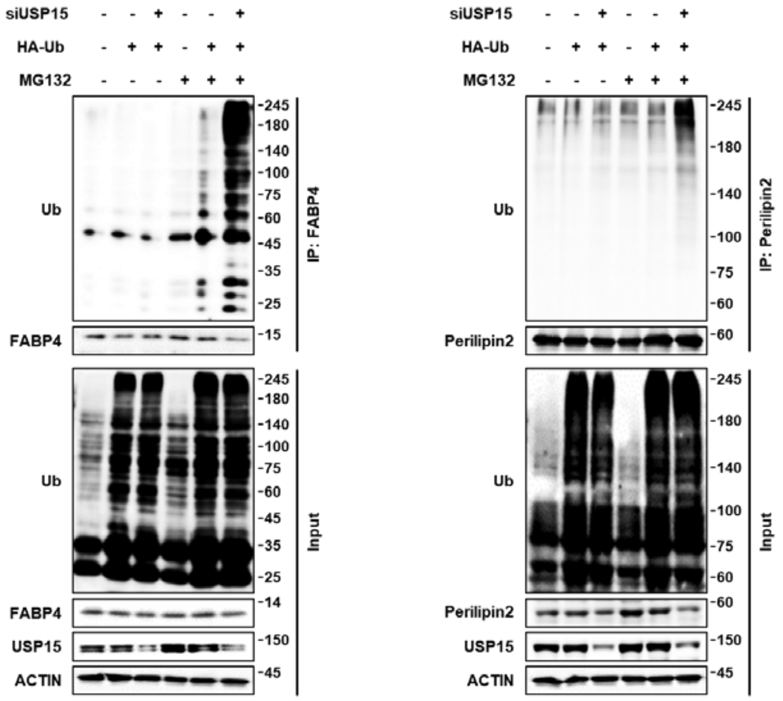

Supplementary Fig. 7. FABP4 and Perilipin2 are de-ubiquitinated by USP15. Western bots of de-ubiquitination assays of FABP4 and Perilipin2. AML12 cells were transiently transfected with HA-Ub or siRNA against USP15 with or without 20 μM MG132 for 8 h.

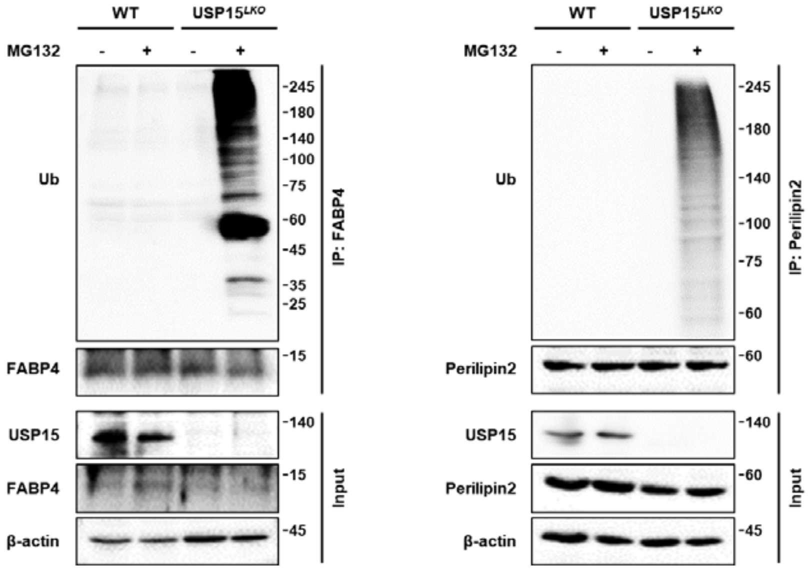

Supplementary Fig. 8. De-ubiquitination of FABP4 and Perilipin2 mediated by USP15 in primary hepatocytes. Primary hepatocytes were isolated and treated with M132. De-ubiquitination assays for FABP4 or Perilipin2 were analyzed by Western blots.

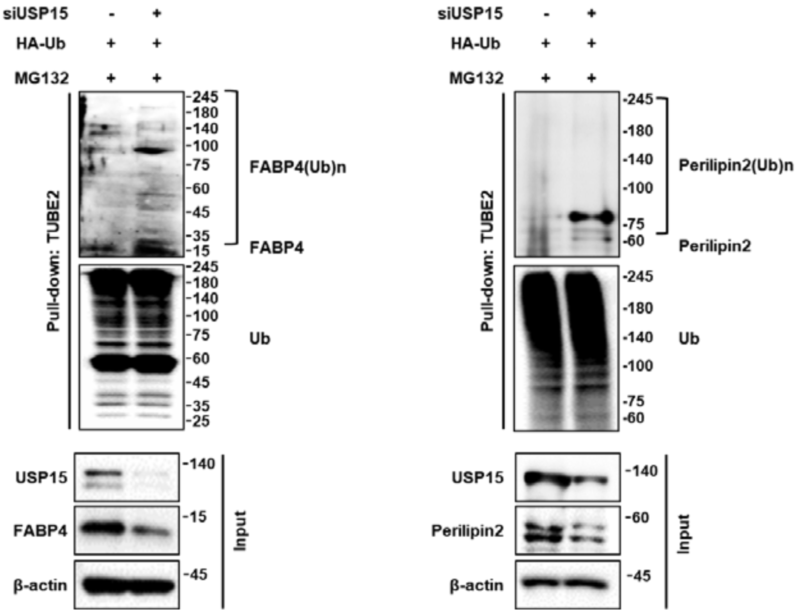

Supplementary Fig. 9. De-ubiquitination of FABP4 and Perilipin2 mediated by USP15. HEK293 cells were transiently transfected with siRNA against USP15. Total ubiquitinated proteins were affinity-purified using TUBE2-agarose pull-down assay. Pulled down total proteins were analyzed by Western blots.

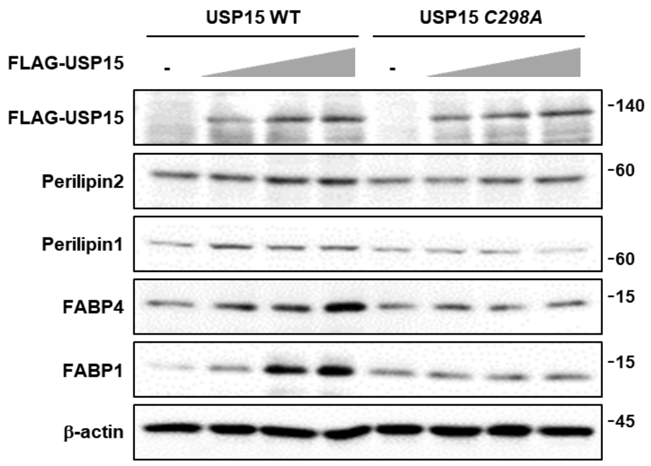

Supplementary Fig. 10. USP15 WT, not USP15 dominant mutant, increased protein levels of endogenous FABPs and perilipins. Effects of USP15 WT or C298A mutant on the endogenous protein levels of FABPs and perilipins. HEK293 cells were over-expressed with either wild-type or mutant form of FLAG-USP15 for 48 h. Protein expression levels were normalized by β-actin.

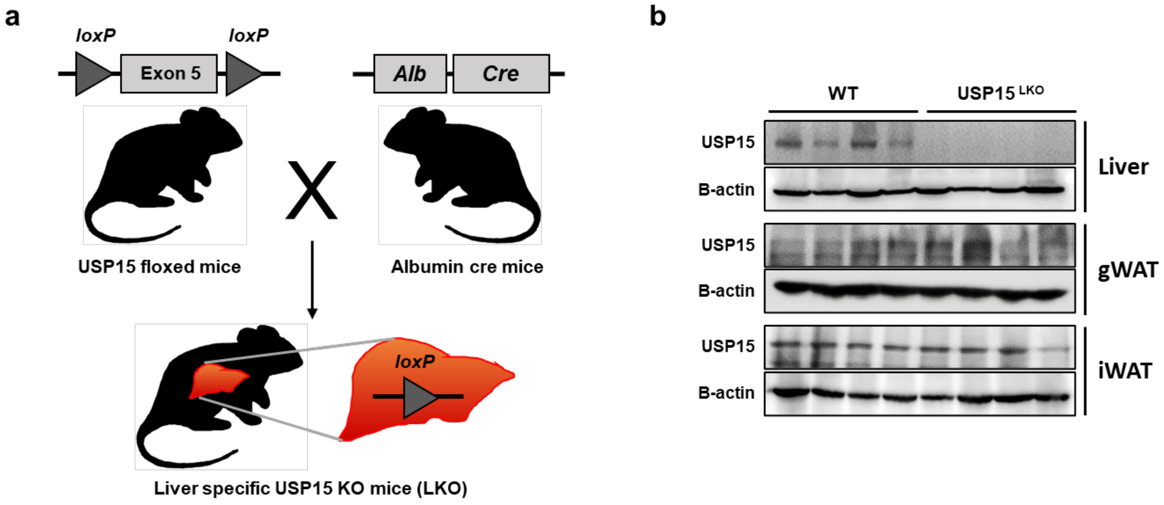

Supplementary Fig. 11. Generation of liver-specific USP15 knockout mice. (a) Schematic workflow showing the establishment of liver-specific USP15 knockout mice. (b) Representative western blot analysis of USP15 expression in the livers, gWAT, or iWAT of wild type (n=4) or *USP15*^{LKO} mice (n=4). Protein expression was normalized to β-actin levels.

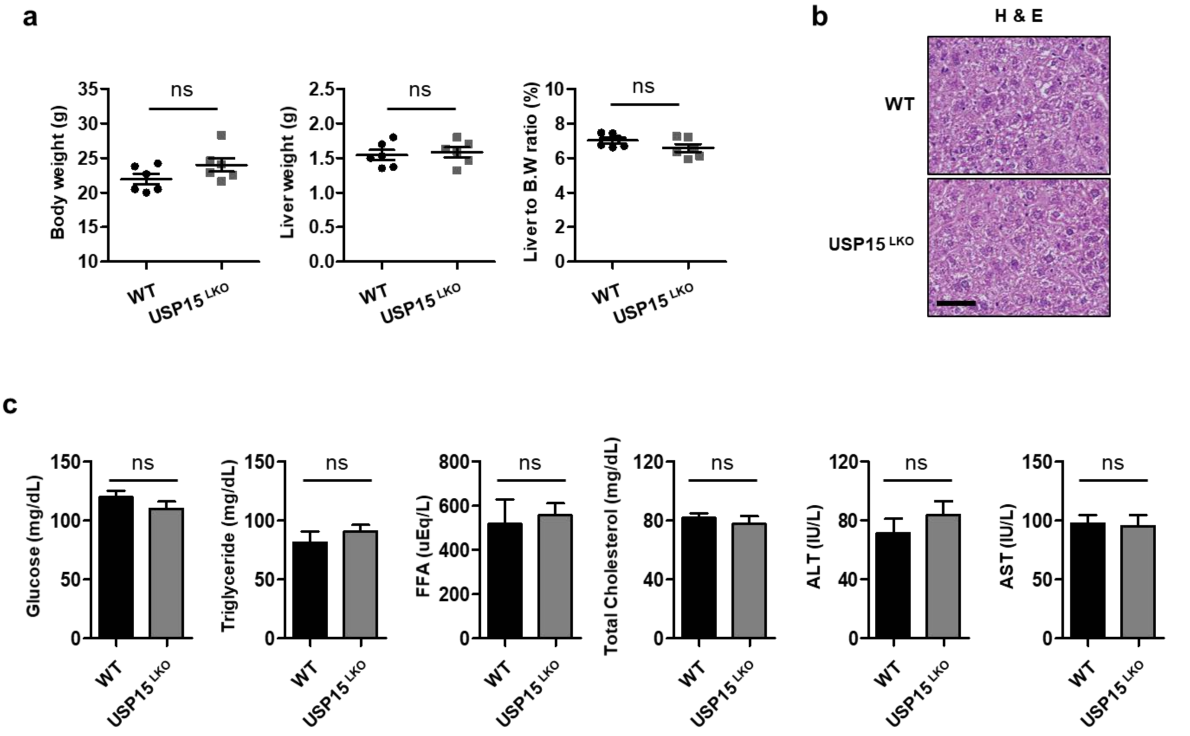

Supplementary Fig. 12. **Ablation of liver-specific USP15 does not affect metabolic phenotype in normal-fat diet fed mice.** (a) Body weight, liver weight, and the ratio of liver weight to the body weight of wild-type and *USP15^LKO^* mice fed an NFD (n=6/group). (b) Slide sections of the liver from wild-type and *USP15^LKO^* mice fed an NFD. Liver sections were stained with H&E. Scale bar indicated 100 μm. (c) Concentrations of glucose, triglyceride, free fatty acid, total cholesterol, ALT, and AST in serum from wild-type and *USP15^LKO^* mice fed an NFD (n=4/group).

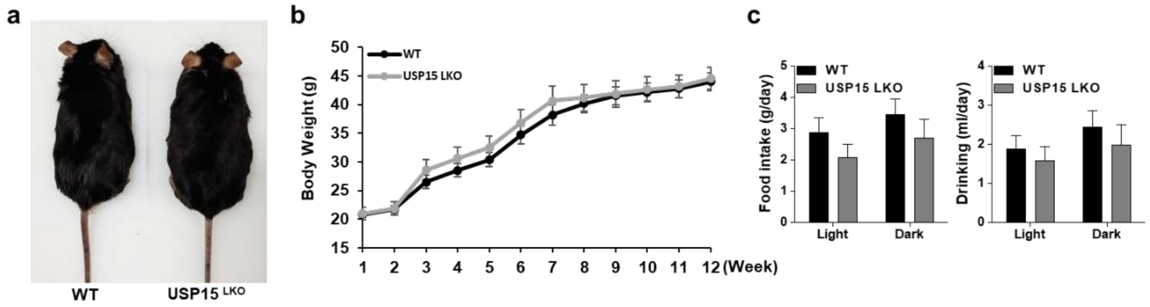

Supplementary Fig. 13. Ablation of liver-specific USP15 does not affect body weight in high-fat diet induced obesity. Both wild-type and *USP15*^{LKO} mice were fed a high-fat diet (HFD) for 12 weeks. (a) Macroscopic view of wild-type and *USP15*^{LKO} mice fed a HFD. (b) Body weight of wild-type and *USP15*^{LKO} mice fed a HFD. (c) Food intake and drinking of wild-type and *USP15*^{LKO} mice fed a HFD.

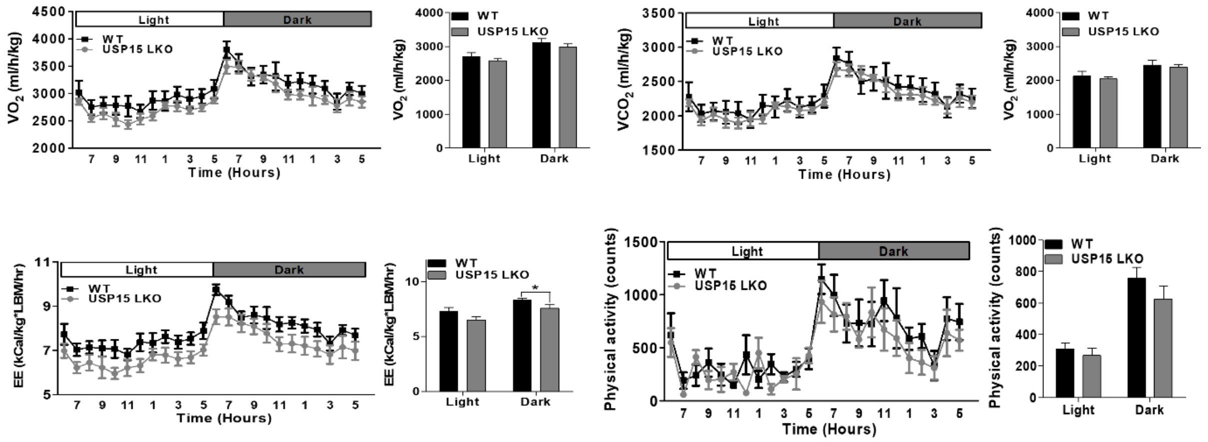

Supplementary Fig. 14. Ablation of liver-specific USP15 does not affect high-fat diet induced obesity. Both wild-type and *USP15*^{LKO} mice were fed a high-fat diet (HFD) for 12 weeks. Metabolic parameters of wild-type and *USP15*^{LKO} mice fed a HFD (n=6/group). The metabolic parameters were measured for 1weeks using a metabolic cage.

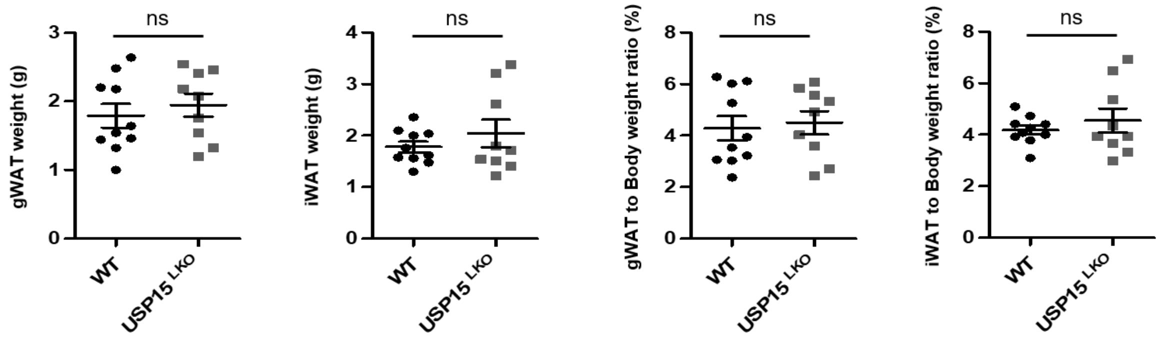

Supplementary Fig. 15. Ablation of liver-specific USP15 does not affect fat mass in high-fat diet fed mice. Both wild-type and *USP15*^{LKO} mice were fed a high-fat diet (HFD) for 12 weeks. gWAT and iWAT weight and their corresponding weight to body weight ratio from high-fat diet mice of wild type (n=10) or *USP15*^{LKO} (n=9).

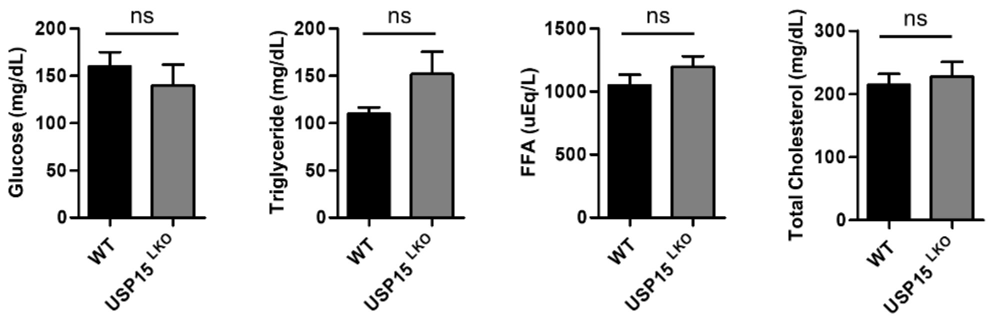

Supplementary Fig. 16. Ablation of liver-specific USP15 does not improve blood metabolic parameters in high-fat diet fed mice. Both wild-type and *USP15*^{LKO} mice were fed a high-fat diet (HFD) for 12 weeks. Concentrations of glucose, triglyceride, free fatty acid, and total cholesterol in serum from wild-type and *USP15*^{LKO} mice fed a HFD (n=4/group)

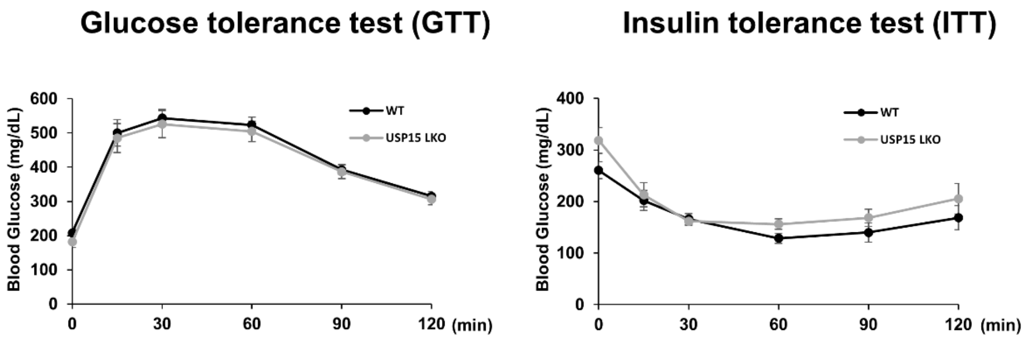

Supplementary Fig. 17. Ablation of liver-specific USP15 does not improve glucose clearance and insulin sensitivity in high-fat diet fed mice. Both wild-type and *USP15*^{LKO} mice were fed a high-fat diet (HFD) for 12 weeks. Results from a glucose tolerance test (GTT) and an insulin tolerance test (ITT) in high-fat diet wild type or *USP15*^{LKO} mice (n=5/group).

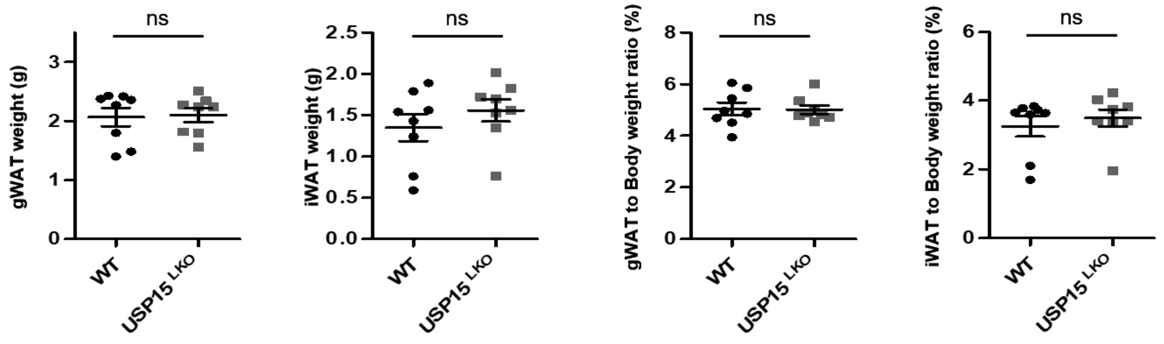

Supplementary Fig. 18. Ablation of liver-specific USP15 does not affect fat mass in FPC diet fed mice. Both wild-type and *USP15^{LKO}* mice were fed a fructose-palmitate-cholesterol (FPC) diet for 16 weeks. gWAT and iWAT weight and their corresponding weight to body weight ratio from FPC diet mice of wild type or *USP15^{LKO}* (n=8/group).

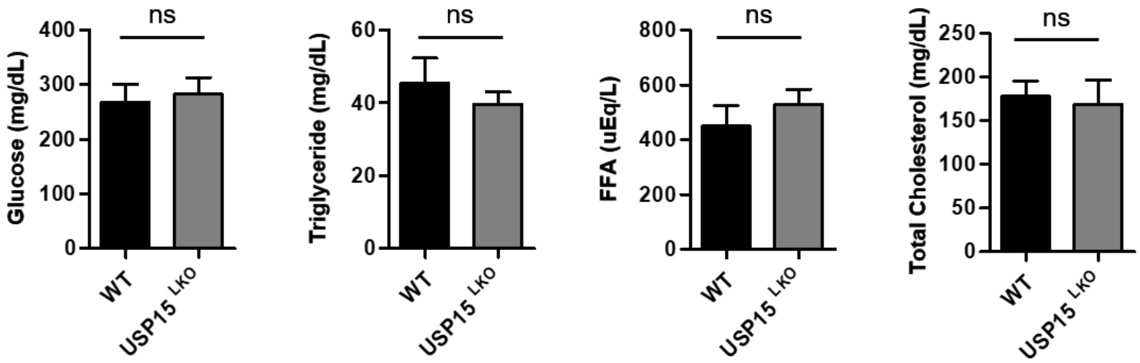

Supplementary Fig. 19. Ablation of liver-specific USP15 does not improve blood metabolic parameters in FPC diet fed mice. Both wild-type and *USP15*^{LKO} mice were fed a FPC diet for 16 weeks. Concentrations of glucose, triglyceride, free fatty acid, and total cholesterol in serum from wild-type and *USP15*^{LKO} mice fed an FPC diet (n=5/group)

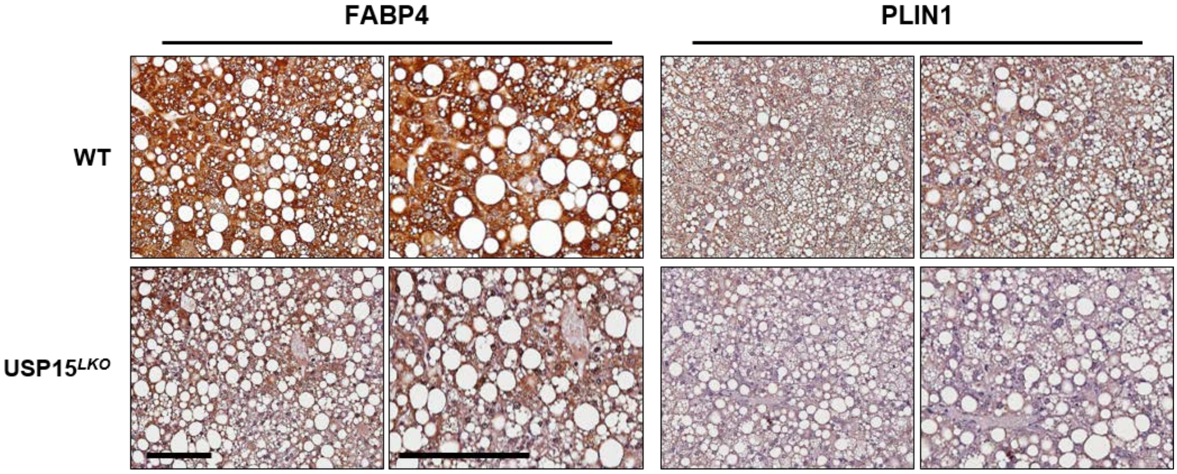

Supplementary Fig. 20. The levels of FABP4 and Perilipin1 were significantly downregulated in the liver of *USP15^{LKO}* mice fed an FPC diet. Slide sections of the liver from wild-type and *USP15^{LKO}* mice fed an FPC diet for 16 weeks. Liver sections were immunohistochemically stained using antibodies against FABP4 and PLIN1. Left panels represent 200 magnifications (x200), and right panels represent 400 magnifications (x 400). Scale bar indicated 100 μm.

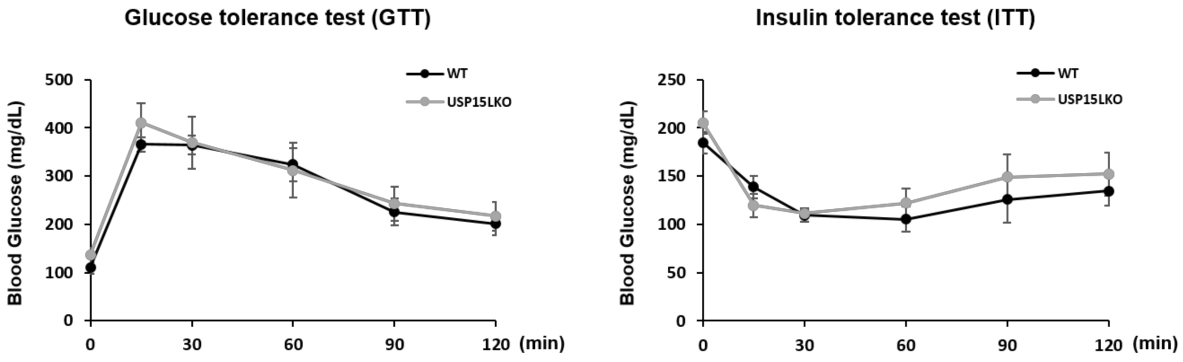

Supplementary Fig. 21. Ablation of liver-specific USP15 does not improve glucose clearance and insulin sensitivity in FPC diet fed mice. Both wild-type and *USP15*^{LKO} mice were fed an FPC diet for 16 weeks. Results from a glucose tolerance test (GTT) and an insulin tolerance test (ITT) in FPC diet wild type or *USP15*^{LKO} mice (n=5/group).

Supplementary Table 1. Quantitative PCR Primer list

Gene Symbol	Forward	Reverse
Human USP15	TCAAAGATGGTGATGCCCAGT	CCCTGTTCAACCACCTTTCG
Human ACTB	CCACCATGTACCCTGGCATT	ACTCCTGCTTGCTGATCCAC
Mouse USP15	GGGGTCCCTCTACTCCTAAGT	TATAAGCAGTATATGACGGGAGACA
Mouse FABP1	GGAAGGACATCAAGGGGGTG	TCACCTTCCAGCTTGACGAC
Mouse FABP4	CATCAGCGTAAATGGGGATT	TCGACTTTCCATCCCACTTC
Mouse PLIN1	TGGATGGAGACCTCCCTGAG	GCACCCTGTACACCCTTCTC
Mouse FASN	TGGGTTCTAGCCAGCAGAGT	ACCACCAGAGACCGTTATGC
Mouse SCD1	GTACCGCTGGCACATCAACT	AAGCCCAAAGCTCAGCTACTC
Mouse CD36	TGATACTATGCCCGCCTCTCC	TTTCCCACACTCCTTTCTCCTCTA
Mouse SREBP1	GATCAAAGAGGAGCCAGTGC	TAGATGGTGGCTGCTGAGTG
Mouse G6PC	CCTGAGGTACCAAGGGAGGA	GAAGGCGTTCCTCAGGTCAG
Mouse ACC1	ATGCGATCTATCCGTCGGTG	TCCTCCAGGCACTGGAACAT
Mouse ACLY	GAAGCTGACCTTGCTGAACC	CTGCCTCCAATGATGAGGAT
Mouse DGAT1	TTCCGTCCAGGGTGGTAGTG	ATGGCACCTCAGATCCCAGTA
Mouse CIDEA	CATACATCCAGCTCGCCCTT	CGTAACCAGGCCAGTTGTGA
Mouse PPARα	TGAGGAAGCCGTTCTGTGAC	CACAATCCCCTCCTGCAACT
Mouse F4/80	CGTCAGCCGATTTGCTATCT	CGGACTCCGCAAAGTCTAAG
Mouse CCL2	TAAAAAACCTGGATCGGAACCAA	GCATTAGCTTCAGATTTACGGGT
Mouse CCL3	GTGACTCACCTTGTGGTCCT	AGGGCAGATCCCAATTGTCAG
Mouse TNFα	CGTCAGCCGATTTGCTATCT	CGGACTCCGCAAAGTCTAAG
Mouse TGFB	CCTGCAAGACCATCGACATG	TGTTGTACAAAGCGAGCACC
Mouse ACTA2	TCACCATTGGAAACGAACGC	CCCCTGACAGGACGTTGTTA
Mouse ACTB	GGCTGTATTCCCCTCCATCG	CCAGTTGGTAACAATGCCATGT
